# *Toxoplasma* ceramide synthases: Gene duplication, functional divergence, and roles in parasite fitness

**DOI:** 10.1096/fj.202201603RRR

**Published:** 2023-10-05

**Authors:** Zisis Koutsogiannis, John G. Mina, Christin A. Albus, Matthijs A. Kol, Joost C. M. Holthuis, Ehmke Pohl, Paul W. Denny

**Affiliations:** ^1^ Department of Biosciences Durham University Durham UK; ^2^ Molecular Cell Biology Division, Department of Biology/Chemistry University of Osnabrück Osnabrück Germany; ^3^ Department of Chemistry Durham University Durham UK

## Abstract

*Toxoplasma gondii* is an obligate, intracellular apicomplexan protozoan parasite of both humans and animals that can cause fetal damage and abortion and severe disease in the immunosuppressed. Sphingolipids have indispensable functions as signaling molecules and are essential and ubiquitous components of eukaryotic membranes that are both synthesized and scavenged by the Apicomplexa. Ceramide is the precursor for all sphingolipids, and here we report the identification, localization and analyses of the *Toxoplasma* ceramide synthases *Tg*CerS1 and *Tg*CerS2. Interestingly, we observed that while *Tg*CerS1 was a fully functional orthologue of the yeast ceramide synthase (Lag1p) capable of catalyzing the conversion of sphinganine to ceramide, in contrast *Tg*CerS2 was catalytically inactive. Furthermore, genomic deletion of *Tg*CerS1 using CRISPR/Cas‐9 led to viable but slow‐growing parasites indicating its importance but not indispensability. In contrast, genomic knock out of *Tg*CerS2 was only accessible utilizing the rapamycin‐inducible Cre recombinase system. Surprisingly, the results demonstrated that this “pseudo” ceramide synthase, *Tg*CerS2, has a considerably greater role in parasite fitness than its catalytically active orthologue (*Tg*CerS1). Phylogenetic analyses indicated that, as in humans and plants, the ceramide synthase isoforms found in *Toxoplasma* and other Apicomplexa may have arisen through gene duplication. However, in the Apicomplexa the duplicated copy is hypothesized to have subsequently evolved into a non‐functional “pseudo” ceramide synthase. This arrangement is unique to the Apicomplexa and further illustrates the unusual biology that characterize these protozoan parasites.

## Graphical abstract

The *Toxoplasma* “pseudo” ceramide synthase (*Tg*CerS2) defines a novel apicomplexan class of these essential enzymes.

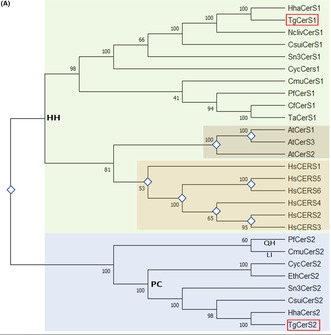

**Abbreviations**
CerS: ceramide synthase
DiCre: dimerizable Cre recombinase
ER: endoplasmic reticulum
EYFP: enhanced yellow fluorescent protein
GFP: green fluorescent protein
GSLs: glycosphingolipids
HFFs: human foreskin fibroblasts
HXGPRT: hypoxanthine–xanthine–guanine phosphoribosyl transferase
IPC: inositol phosphorylceramide
KDS: keto‐di‐hydro‐sphingosine
Ki. Red: killer red
Lag1p: longevity‐assurance gene
Lac1c: longevity‐assurance gene
LDL: low‐density lipoprotein
MPA: mycophenolic acid
PV: parasitophorous vacuole
SM: sphingomyelin
Sph: sphinganine
NDB Sph: NBD sphinganine
SPT: serine palmitoyltransferase
*Tg*CerS1: *Toxoplasma gondii* ceramide synthase 1
*Tg*CerS2: *Toxoplasma gondii* ceramide synthase 2
TE: Toxoplasmic encephalitis
TDMs: transmembrane domains
UTR: untranslated region

## 1 INTRODUCTION

The Apicomplexa is a phyla of infectious protozoa comprising pathogens of both domestic animals and humans.^1^ These include the haemosporidian *Plasmodium* spp., including *P. falciparum* the causative agent of severe malaria; the coccidian *Cryptosporidium* spp. (diarrhea), *Eimeria* spp. (coccidiosis in poultry and cattle) and *Toxoplasma gondii* (toxoplasmosis).^2^ *Toxoplasma* is an obligate intracellular parasite with the ability to invade and colonize a broad range of nucleated cells from warm‐blooded vertebrates; therefore, toxoplasmosis is one of the most prevalent infections and is estimated to affect 2–3 billion people worldwide.^3^ Importantly, infection is a significant cause of congenital defects and subsequent abortions in both economically important domestic animals^4^ and humans.^5^ However, treatment regimens are limited and have remained largely unchanged since the 1950s,^6^ and there are no therapies available for chronic infection with encysted bradyzoite forms.^7^ To this end efforts are focused on identifying new drug targets. Recent studies have shown major differences in the lipid, particularly sphingolipid, profiles of apicomplexans compared to the host.^3^, ^8^, ^9^ Sphingolipids are ubiquitous amphipathic plasma membrane lipids involved in a myriad of signaling processes in all eukaryotes, including *Toxoplasma*,^10^ and their biosynthesis has previously been validated as a drug target against the kinetoplastid protozoan parasites *Leishmania* ^11^, ^12^, ^13^ and *Trypanosoma*,^14^ as well as fungal pathogens.^15^, ^16^, ^17^ In proliferative tachyzoite infection sphingolipids can be scavenged from the host^8^, ^18^, ^19^ and *Toxoplasma* have been shown to possess sphingomyelin, the primary mammalian phosphosphingolipid.^3^ However, they have also been demonstrated to harbor a relatively high level of ethanolamine phosphorylceramide, which is only found as a trace phosphosphingolipid in the host cell,^3^ and non‐mammalian inositol phosphorylceramide.^8^ In addition, host sphingolipid biosynthesis is non‐essential for *Toxoplasma* proliferation,^8^, ^19^ and the parasite retains a fully functional biosynthetic pathway^8^, ^18^, ^20^ which is active throughout its lifecycle.^21^ This indicated that de novo synthesis is important for parasitism and a potential target for therapeutic intervention.

Ceramide, the product of ceramide synthase (CerS or Lag1p [longevity‐assurance gene] in yeast), is the central molecule among the sphingolipid precursors which functions in a plethora of biological processes, including apoptosis, growth arrest, and stress responses.^22^ The first, rate‐limiting stage in sphingolipid biosynthesis is mediated by serine palmitoyltransferase (SPT), which catalyzes the condensation of L‐serine and, typically, palmitoyl‐CoA to form 3‐keto‐di‐hydro‐sphingosine (KDS or 3‐keto‐sphinganine)^23^ in the endoplasmic reticulum (ER). Subsequently, KDS is reduced and then *N*‐acylated by CerS to form ceramide, the central metabolic hub of sphingolipid biosynthesis, which is then transported to the Golgi apparatus where it is converted into glycosphingolipids (GSLs) and, in mammals, sphingomyelin (SM).^24^, ^25^

**FIGURE 1:**
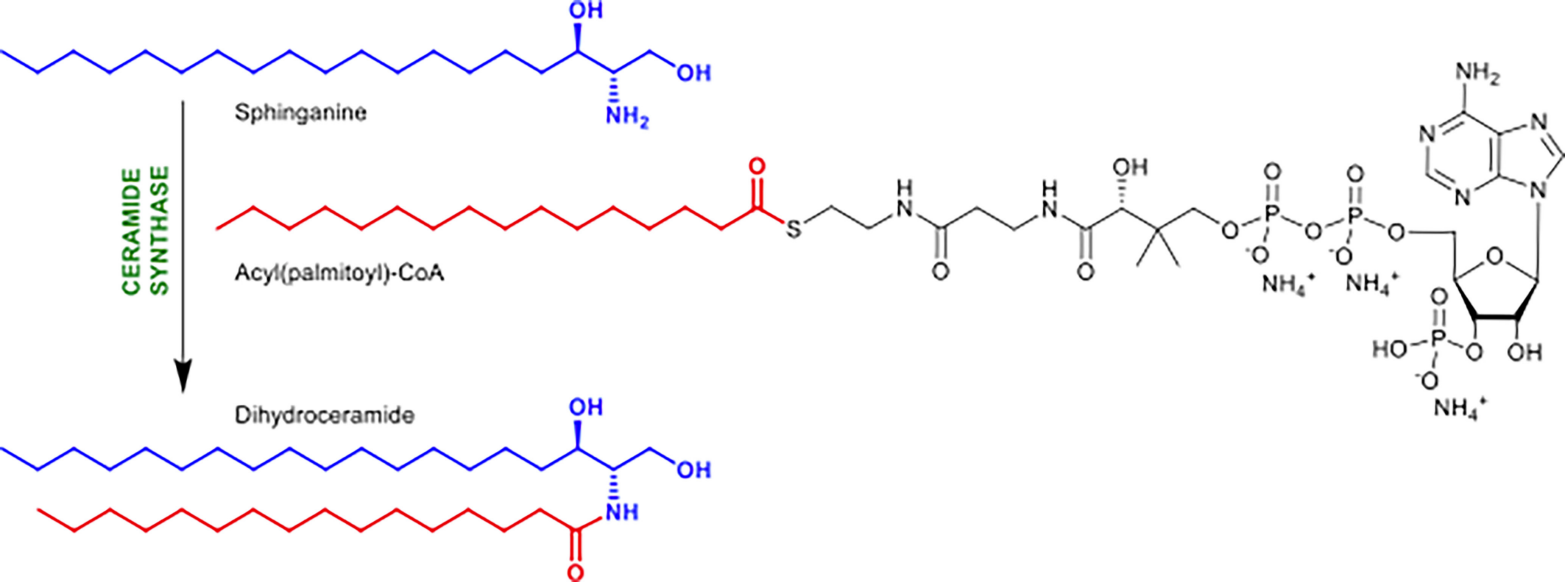
Substrates and products of ceramide synthase.

Although *Toxoplasma* and the other apicomplexans maintain this pathway, it is in many ways dramatically divergent when compared to the mammalian cell machinery.^22^ For example, the first functionally characterized enzyme in the apicomplexan biosynthetic pathway was a functional orthologue of the yeast inositol phosphorylceramide (IPC) synthase, an enzyme with no mammalian equivalent.^8^, ^26^ Furthermore, the apicomplexan SPT is, uniquely in the Eukaryota, of bacterial origin and presumed to have been acquired via lateral gene transfer.^20^ In addition, both orthologues of the *Toxoplasma* SPT (1 and 2) have recently been demonstrated to be involved ceramide regulation in vitro and in both acute and chronic in vivo infection.^27^ However, the characterization of the apicomplexan CerS, the second key step in eukaryotic sphingolipid biosynthesis (Figure 1), remained absent. Here, we describe and characterize the *Toxoplasma Tg*CerS1 and *Tg*CerS2, which also have orthologues in the wider Apicomplexa. Utilizing a combination of biochemical, genetic and phylogenetic tools we demonstrate that while *Tg*CerS1 is a functional CerS, *Tg*CerS2 lacks this activity in vitro. However, while functional *Tg*CerS1 plays little role in parasite fitness, *Tg*CerS2 was indicated to have a particularly important role. Phylogenetic analyses indicated that the apicomplexan “pseudo” ceramide synthase, *Tg*CerS2 and orthologues, may be the result of an ancient gene duplication event and a subsequent divergence that is unique to the Apicomplexa.

## 2 MATERIALS AND METHODS

### 2.1 Culturing of *Toxoplasma gondii* and host cells

Human foreskin fibroblasts (HFFs; SRC‐1041, ATCC®) were cultivated in culture‐treated plastics (T‐75 s; T‐25 s and 6 well plates) in the presence of Dulbecco's modified Eagle's medium (DMEM‐Gibco) supplemented with 10% fetal bovine serum (Sigma‐Aldrich), 2 mM L‐glutamine and 1% penicillin–streptomycin solution. HFF cells were not used beyond passage 20. All strains of *Toxoplasma gondii* including RH.diCre.Δku80^28^ (a kind gift from Markus Meissner, LMU Munich, Germany), RH.ΔKu80.ΔCerS1, RH.diCre.Δku80:CerS1 and RH.diCre.Δku80:CerS2 were maintained in vitro by serial passages on monolayers of HFFs maintained at 37°C, 5% CO_2_ in a humidified incubator. Freshly egressed parasites were assessed for viability by Trypan blue (0.4% w/v) to ensure high viability (≥95%) before downstream experiments.

### 2.2 Isolation of *Toxoplasma* genomic DNA and RNA

Genomic DNA was extracted from *T. gondii* tachyzoites to use as PCR template by pelleting parasites and resuspending in PBS. DNA extraction was then performed using the QIAamp DNA Blood Mini Kit (Qiagen) as per the manufacturer's protocol. RNA was isolated from freshly purified tachyzoites of *Toxoplasma gondii* using RNeasy Micro Kit (Qiagen) and subsequently reverse‐transcribed into first‐strand cDNA using Qiagen One Step RT‐PCR Kit System.

### 2.3 Cell‐free expression *Tg*CerS1 and *Tg*CerS2

*Tg*CerS1 and *Tg*CerS2 open reading frames were synthesized (GenScript) and following PCR amplification using primer pairs *Tg*CERS1.CFE.P1/*Tg*CERS1.CFE.P2_FLAG and *Tg*CERS2.CFE.P1/*Tg*CERS2.CFE.P2_FLAG, respectively (Table S1). In‐Fusion (TaKaRa) cloned into pEU‐E01‐MCS. The mammalian pCMV‐Tag2B‐CerS2/CerS5 expression vectors were a kind gift from Tony Futerman, Weizmann Institute, Israel. HsCerS2‐V5‐His and FLAG‐HsCerS5‐V5‐His were PCR‐amplified and cloned into the pEU Flexi vector^29^ (a kind gift from James Bangs, University at Buffalo, New York). To this end, a KpnI restriction site was first introduced directly between the hSMS1 ORF and V5 tag in the pEU‐Flexi vector using site‐directed mutagenesis according to the Stratagene Quick Change protocol with primers pEU.Flexi.P1 and pEU.Flexi.P2 (Table S1). HsCerS2‐V5‐His and FLAG‐HsCerS5‐V5‐His were then PCR‐amplified using primer pairs *Hs*CERS2.CFE.P1/*Hs*CERS2/5.CFE.P2 and *Hs*CERS5.CFE.P1/*Hs*CERS2/5.CFE.P2 respectively (Table S1), the products ligated into the pJET1.2 blunt vector (Thermo Scientific), digested with XhoI and KpnI alongside with the pEU‐KpnI Flexi acceptor vector and the final constructs were obtained by T4 ligation (Invitrogen). Using the Protein Research Kit S16 (CellFree Sciences) cell‐free expression of *Tg*CerS1, *Tg*CerS2, *Hs*CerS2‐V5‐His, and FLAG‐*Hs*CerS5‐V5‐His were performed as described for sphingomyelin synthases^30^ but without the application of a dialysis reservoir and in the presence of 100 nm diameter liposomes consisting of PC:PE:PI 2:2:1 (mol:mol:mol). Protein expression was confirmed by SDS‐PAGE and western blotting using ready‐made gels (Invitrogen; 100 V, 60 min) before transferring to a nitrocellulose membrane (Life Technologies; Bio‐Rad; 100 V for 60 min). Following blocking (2 mM Tris–HCl, 50 mM NaCl [pH 7.5], 0.5 mL Tween 20 and 5% w/v fatty‐acid‐free BSA for 60 min at room temperature), protein expression was determined using primary anti‐FLAG or anti‐V5 tag antibodies (AbCam; 1:500, overnight at 4°C) and rabbit anti‐mouse IgG (H + L) secondary HRP‐conjugated antibody (ThermoFisher; 1:5000, 60 min at 4°C). Detection was achieved with Pierce™ ECL Western Blotting Substrate using a ChemDoc XR+ (Bio‐Rad).

### 2.4 Ceramide synthase assay

Following determination of expression, equivalent concentrations of *Tg*CerS1 and *Tg*CerS2, plus controls, were assayed for ceramide synthase activity in LoBind tubes (Eppendorf) as follows. A 100 μL reaction volume with 80 μL of proteoliposome mixture, 15 μL cell‐free expression translation buffer (15 mM HEPES‐KOH [pH 7.8], 50 mM potassium acetate, 1.25 mM magnesium acetate, 0.2 mM spermidine hydrochloride, 0.6 mM ATP, 0.125 mM GTP and 8 mM creatine phosphate), 1 μL NBD‐Sph (5 mM stock; Avanti Polar Lipids Inc.), 1 μL Acyl‐CoA C16:0/C24:0 (5 mM stocks; Avanti Polar Lipids Inc.), and 4 μL delipidated BSA (5 μM stock; Sigma‐Aldrich). Following incubation (60 min at 37°C) the reaction was halted by addition of 375 μL chloroform: methanol (1:2) and lipid extracted according to Bligh and Dyer. Following drying and re‐suspension in 10:10:3 chloroform:methanol:H_2_O, lipid extracts were transferred to a NANO‐ADAMANT HP‐TLC plate (Macherey & Nagel) using an ATS5 TLC sampler (CAMAG, Berlin, Germany). The TLC was developed in CHCl_3_:MeOH:2M_aq_NH_4_OH (40:10:1 v:v:v) or CHCl_3_:MeOH:H_2_O (80:12:1 v:v:v) in a CAMAG ADC2 automatic TLC developer.^31^ The NBD lipids were detected using a Typhoon FLA 9500 biomolecular imager (GE Healthcare Life Sciences) operated in Cy2 fluorescence mode with 473 nm excitation laser, BPB1 filter, 50 μm pixel size, and PMT voltage setting of 290 V. Data were processed using ImageLab (Bio‐Rad Laboratories) to adjust intensity.

### 2.5 Cloning and subcellular localization of *Tg*CerS1 and *Tg*CerS2

Primers were designed to amplify and FLAG tag the *Tg*CerS1 and *Tg*CerS2 coding sequences: *Tg*CerS1Xpress/*Tg*CerS1FLAGXpress_R and *Tg*CerS2Xpress_F/*Tg*CerS2FLAGXpress_R from the synthetic genes (Table S2). The resultant PCR products were introduced into NheI (NEB) linearized ToxoXpress vector (pTXP; designed, created, and validated in‐house; Figure S1) using the In‐Fusion HD cloning kit (TaKaRa) as per manufacturer's protocol (Table S3) to create pTXP_*Tg*CerS1‐FLAG and pTXP_*Tg*CerS2‐FLAG. All constructs were verified by sequencing (Eurofins Genomics) prior to further work. Transient transfections were carried out using a 4D Nucleofector (Lonza), protocol FI 158. Briefly, parasites freshly lysed from HFFs monolayer were homogenized by passage through a 25‐gauge needle and isolated by centrifugation at 1500 × *g* for 10 min at 4°C. The pellet was resuspended in P3 buffer with added supplementary buffer P1 (Lonza). 20 μL of the parasite suspension (10^7^ mL^−1^) were added to a dried pellet of ethanol‐precipitated pTXP_*Tg*CerS1‐FLAG or pTXP_*Tg*CerS2‐FLAG plasmid and electroporated. Subsequently, 100 μL of media was added, and 10 μL or 20 μL were transferred to 24‐well plates containing confluent HFF cells grown on glass coverslips before incubation at 37°C, 5.0% CO_2_. Cells were fixed with 4% paraformaldehyde in PBS (pH 7.4) for 15 min and then permeabilized with 0.4% (v/v) Triton X‐100 in PBS for 10 min, before incubation in blocking buffer (PBS supplemented with 1% [w/v] BSA (Sigma‐Aldrich), 0.1% fish skin gelatin (Sigma‐Aldrich) and 0.1% [v/v] Triton X‐100) for 15 min at room temperature. Samples were incubated with polyclonal rat anti*‐Tg*SPT1_Δ158 (1:200)^20^, ^27^ and mouse monoclonal anti‐FLAG antibody (1:200; AbCam) in blocking buffer overnight at 4°C. After PBS washing, samples were incubated with Alexa Fluor® 488 anti‐rat and Alexa Fluor® 647 anti‐mouse secondary antibodies (ThermoFisher) at 1:500 in blocking buffer for 1 h at room temperature. The samples were incubated with Hoechst 33342 (Sigma‐Aldrich) in PBS for 10 min, mounted using Vectashield H‐1000 (Vector labs) and sealed before imaging.

### 2.6 Construction of plasmids for CRISPR/Cas9‐based manipulation

CRISPR/Cas‐9 was utilized to accelerate the generation of the conditional Δ*Tg*CerS1 and Δ*Tg*CerS2 lines and also to create CRISPR/Cas‐9 KOs. All CRISPR/Cas9 plasmids designed and used in this study were created by using Q5 Site‐Directed Mutagenesis Kit (NEB‐ E0554S) based on the previously described CRISPR/Cas9 plasmid^32^ as primary scaffold, as per the manufacturer's protocol. All constructs were verified by sequencing (Eurofins Genomics) prior to further work. Primers for *Tg*CerS1 and *Tg*CerS2 sgRNAs were predicted and designed using online tools including EuPaGDT, E‐CRISP, and CHOPCHOP (Table S4), targeting the 5′ and 3′ of the respective coding regions to ensure efficient gene deletion and subsequent DNA insertion. CRISPR/Cas‐9 plasmids were transfected along with supplementary DNA cassettes to allow mutant verification and positive selection. In the case of non‐conditional knockouts, a GFP reporter gene was introduced to the corresponding genes *Tg*CerS1 and *Tg*CerS2, respectively, after DNA amplification from a previously described vector^33^ using designed primers (Table S5).

### 2.7 Construction of cassettes for inducible recombinase‐based knockout

A pUC19 (NEB) plasmid was used as a backbone for the constructs after it was linearized with FD PvuII to customize the subsequent assembly of the appropriate fragments. All DNA amplicons for plasmids constructs were amplified with high fidelity Phusion Polymerase (NEB) to minimize replication errors. pUC19‐5′UTR‐loxP‐*Tg*Cers1‐2A‐KillerRed‐loxP‐EYFP‐3′UTR‐5′UTR‐HXGPRT‐3′UTR and pUC19‐5′UTR‐loxP‐*Tg*CerS2‐2A‐KillerRed‐loxP‐EYFP‐3′UTR‐5′UTR‐HXGPRT‐3′UTR were designed to be knocked into the *Tg*CerS1 or *Tg*CerS2 loci respectively. Briefly, four different DNA fragments were amplified using primers sets F01, F02, F03, and F04 (Tables S6 and S7; Integrated DNA Technologies [IDT]). Fragments were cloned into a PvuII linearized pUC19 using In‐Fusion HD cloning kit (TaKaRa) as per manufacturer's instructions. All constructs were verified by sequencing (Eurofins Genomics) prior to further work. FastDigest (FD) LguI and a combination of FD SacI/FD Ajul (for *Tg*Cers1) and FD XbaI and combination of FD ScaI and FD Hind III (for *Tg*Cers2) were used to digest plasmids at 37°C for 30 min and electrophoresed on a 0.8% agarose gel to verify successful assembly. Finally, FD SphI and FD CpoI were used to linearize *Tg*CerS1 DNA cassette and FD HindIII was used to linearize *Tg*CerS2 DNA cassette before electroporation into freshly egressed RH.diCre (parental) parasites as described above.

### 2.8 *Toxoplasma* mutant strain selection and verification

All transfections were performed by a 4D Nucleofector (Lonza) as described above. Transfected parasites were incubated overnight at 37°C and 5% CO_2_ with media being replaced with complete DMEM containing 25 μg/mL MPA (Sigma‐Aldrich) and 50 μg/mL xanthine (Sigma‐Aldrich) the following day and left to grow for at least 8 days with the selection media refreshed every 48 h. Clonal line selection was then performed with newly acquired mutant strains before molecular verification by diagnostic PCR using GoTaq DNA polymerase (Promega) and multiple sets of primers (Figure S2; Tables S8 and S9). In addition, confocal fluorescence microscopy monitoring the red (585/610 nm) to yellow (513/527 nm) fluorescence switch was utilized as described below. CRISPR/Cas‐9 non‐conditional KOs were also assessed microscopically monitoring GFP signal post‐transfection.

### 2.9 Conditional *Tg*CerS1 and *Tg*CerS2 knockouts

*Tg*CerS1 and *Tg*CerS2 conditional knockouts were obtained after the addition of 100 nΜ rapamycin to RH.diCre:CerS1 and RH.diCre:CerS2 strains, respectively. Briefly, tachyzoites were collected from HFFs by scraping cells and passing through a 25G needle by syringe lysis. Following centrifugation at 50 × *g* for 5 min to allow removal of cell debris, the supernatant was centrifuged again at 500 × *g* for 5 min. Parasites were then resuspended and incubated for 4 h at 37°C, 5% CO_2_ in complete DMEM with 100 nM of rapamycin before washing twice with warm media to remove excess rapamycin. Following viability assessment by trypan blue (0.4% w/v), the parasites were then used to infect HHF monolayers. *Toxoplasma* parental line was also tested under the same treatment conditions to exclude rapamycin effects on parasite fitness.

### 2.10 Confocal fluorescence microscopy

*Tg*CerS1‐FLAG, *Tg*CerS2‐FLAG, RH.diCre.Δku80, and RH.diCre:CerS1 and RH.diCre:CerS2 uninduced and rapamycin‐induced parasites, were allowed to invade and replicate in a HHF monolayers grown on glass coverslips as previously described. Fluorescence images were acquired using laser scanning confocal microscope Zeiss LSM 880 with AiryScan equipped with excitation laser 405, Argon 458, 488, 514, He‐Ne 543, 594, and 633 and AiryScan filter set combinations BP 420–480 + BP 495–550, BP 420–480 + BP 495–620, BP 420–480 + LP 605, BP 465–505 + LP 525, BP 495–550 + LP 570, and BP 570–620 + LP 645 (Durham Centre for Bioimaging Technology). For each image, the dynamic range was checked to avoid saturation, except with the Hoechst staining where host cells masked the detection of parasite nuclei at low gain/laser power values. AiryScan images were automatically processed using default values. 40X, 63X, and 100X oil lenses were used to capture red and yellow/green fluorescence. CZi outputs were exported and analyzed using ImageJ and Imaris software.

### 2.11 Phenotypic characterization—*Toxoplasma* fitness

Plaque and monolayer disruption assays were employed to analyze parasite fitness. In both cases, HHF cells were grown to confluence in 24 (plaque) and 6 (disruption) well plates. 100 and 10^5^ parasites per well respectively were added and the plates incubated for 12 days or up to 96 h. Subsequently, wells were gently washed with PBS and fixed with 4% paraformaldehyde (pH 7.4) for 20 min at room temperature. 0.05% w/v Crystal Violet (CV) solution then replaced the fixative and, after 30 min at room temperature, the wells were gently rinsed twice with water and left to dry before coating with 0.3% agarose. Plaques and disruption areas were photographed using an Olympus inverted microscope CKX53 and a 5 MP XCAM camera 1080p, under 4X magnification. ImageJ was then used to determine plaque sizes and monolayer disruption areas. Values are expressed as mean values of three independent experiments ± SD. *P*‐values were calculated using unpaired two‐tailed Student's *t*‐test unless stated otherwise. P‐value significance thresholds were set at: *p < .05, **p < .01, ***p < .001.

### 2.12 Phylogenetic analysis

ToxoDB, VEuPathBD, TriTrypBD, PlasmoDB, UniProt were used in order to identify CerS orthologues in various taxa and species. All accession numbers listed in the text aside from: *E. necatrix* ENH_00074610.1‐p1, *E. maxima* EMWEY_00008780‐t26_1‐p1, *E. mitis* EMH_0037690‐t26_1‐p1, *E. acervuline* EAH_00007580‐t26_1‐p1, and *E. brunetti* EBH_0045800‐t26_1‐p1. Multiple amino acid alignment was performed using MAFFT and MUSCLE alignment algorithms.^34^ The predicted CerS amino acid sequences selected for phylogenetic analyses were aligned using ClustalW,^35^ manually edited to remove non‐aligned regions, and then re‐aligned in ClustalW with the output selected as a ClustalW format. Molecular Evolutionary Genetics Analysis (MEGA‐X)^36^ was used to reconstruct phylogenetic trees and perform gene duplication analysis. Neighbor Joining, UPGMA Tree and Maximum Likelihood methods were used to allow for equal and unequal rates of evolution between the CerS's of different species and taxa. NGphylogeny.fr was also used to visualize MAFFT alignments and identify CerS consensus regions. Sequence accession numbers with respective organism names are listed in Table S10.

## 3 RESULTS

### 3.1 Identification of *Toxoplasma gondii* ceramide synthases

In all eukaryotic systems studied to date, ceramide synthases (CerS) are ER‐resident integral membrane proteins that *N*‐acylate di‐hydrosphingosine (sphinganine) to produce di‐hydroceramide which is then desaturated to form ceramide, the simplest sphingolipid and a key secondary messenger in numerous cellular pathways.^37^ First identified as encoded by the longevity‐assurance genes Lag1p and Lac1p in yeast,^38^ orthologues were subsequently found to be ubiquitous in the Eukaryota. All eukaryotes studied to date have been found to encode at least two CerS orthologues,^39^ with humans expressing six isoforms with each generating ceramides with a defined acyl chain length.^39^, ^40^ All orthologues harbor the conserved 52 amino acid Lag1 motif. Although the precise functionality of this domain remains unclear, 9 of the 11 canonical residues are essential for CerS activity.^41^

**FIGURE 2:**
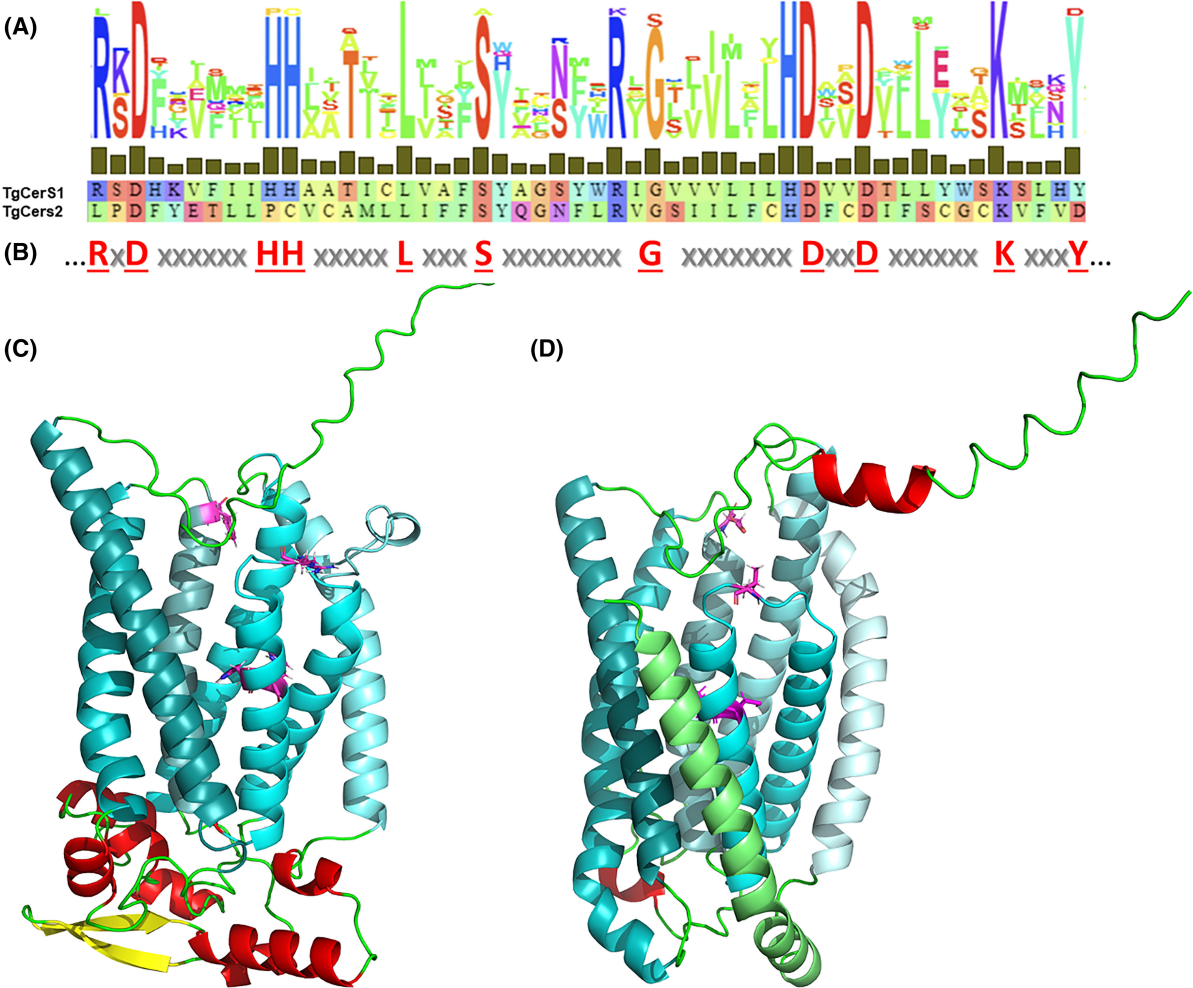
*Tg*CerS1 and *Tg*CerS2 catalytic site residues and predicted structure. (A) Alignment of *Tg*CerS1 (residue 208–259) and *Tg*CerS2 (residue 169–220) putative active site residues with conservation in relation to the Lag1 consensus motif shown above. Hydrophobic amino acids are depicted in green; aromatic amino acids in cyan; aliphatic amino acids in red and orange; and large polar acids in purple and blue.^42^ (B) Lag1 consensus motif, canonical residues depicted in red. Ribbon diagram of the ab‐initio models derived with Alphafold2^43^ of ceramide synthases (C) *Tg*CerS1 and (D) *Tg*CerS2. The transmembrane helixes are shown different shades of cyan from H1 light to H7 dark. Arginine (R208), double histidine (H217 H218), and tyrosine (Y259) residues in *Tg*CerS1, as well as leucine (L168), proline (P178), cysteine (C179), and aspartic acid (D220) residues in *Tg*CerS2, are highlighted in magenta and shown in stick formation. Putative extracellular helices shown in red, strands in yellow and loops in green. In both cases the C‐termini point upwards, toward the cytosol.

An initial search of the *Toxoplasma* genome database (toxodb.org/) revealed a single, putatively encoded 383 amino acid CerS orthologue, TGGT1_316450 (named *Tg*CerS1). However, re‐probing the database with the identified sequence yielded a paralog, TGGT1_283710 (named *Tg*CerS2) a predicted 342 long amino acid protein which exhibited only 16.8% sequence identity and 34.5% similarity to *Tg*CerS1. *Tg*CerS2 demonstrated higher homology within the Lag1 motif region (25% identity and 42% similarity, CLUSTALW2 and MAFFT alignment); however, it lacked the essential and canonical arginine (R208), double histidine (H217 H218) and tyrosine (Y259) residues (Figure 2A,B; *Tg*CerS1 numbering). All of these were subject to non‐conservative changes in *Tg*CerS2, leucine (L169), proline (P178), cysteine (C179) and aspartic acid (D220) respectively (Figure 2A,B; *Tg*CerS2 numbering). While the structure–function relationships of CerSs are not well studied, their topology displays a conserved consensus of seven predicted transmembrane domains (TMDs) with the active site facing the lumen of the ER^44^, ^45^ and their acyl chain preference determined by a region of 11 residues forming an *exo*‐membranous loop.^46^ *Tg*CerS1 and *Tg*CerS2 maintain the consensus of seven TMDs as predicted by the protein structure prediction software AlphaFold2^43^ (Figure 2C,D) and, increasing the confidence in the prediction, RosettaFold^47^ (Figure S3). Notably, while both structure prediction programs revealed the same transmembrane architecture, including the Lag1 motif, the extracellular loop structures were predicted with much lower confidence and differed significantly in secondary structure and overall fold.

The CRISPR/Cas9‐based genome‐wide data generated by Sidik et al., 2016^48^ indicated that both orthologues may have only minor a role in parasite fitness and expression profiling demonstrated their presence throughout the lifecycle.^49^ In addition, utilizing a spatial proteomic approach, hyperplexed localization of organelle proteins by isotope tagging (hyperLOPIT), Barylyuk et al., 2020^50^ indicated that like all studied ceramide synthases, *Tg*CerS2 is located in the ER. However, the localization of *Tg*CerS1 was not assigned (Table 1).

**TABLE 1:** Database information regarding the identified CerS orthologues *Tg*CerS1 (TGGT1_316450) and *Tg*CerS2 (TGGT1_283710).

Gene ID	Phenotype score	Length (aa)	Expression	LOPIT
TGGT1_316450 (*Tg*Cers1)	−1.55	383	Throughout Lifecycle	NA
TGGT1_283710 (*Tg*CerS2)	−1.09	342	Throughout Lifecycle	ER

*Note*: Phenotype scores from Sidik et al., 2016,^48^ expression profiles from Fritz et al., 2012^49^ and hyperLOPIT data from Barylyuk et al., 2020.^50^

Abbreviation: NA, not assigned.

### 3.2 Activity and localization of *Tg*CerS1 and *Tg*CerS2

**FIGURE 3:**
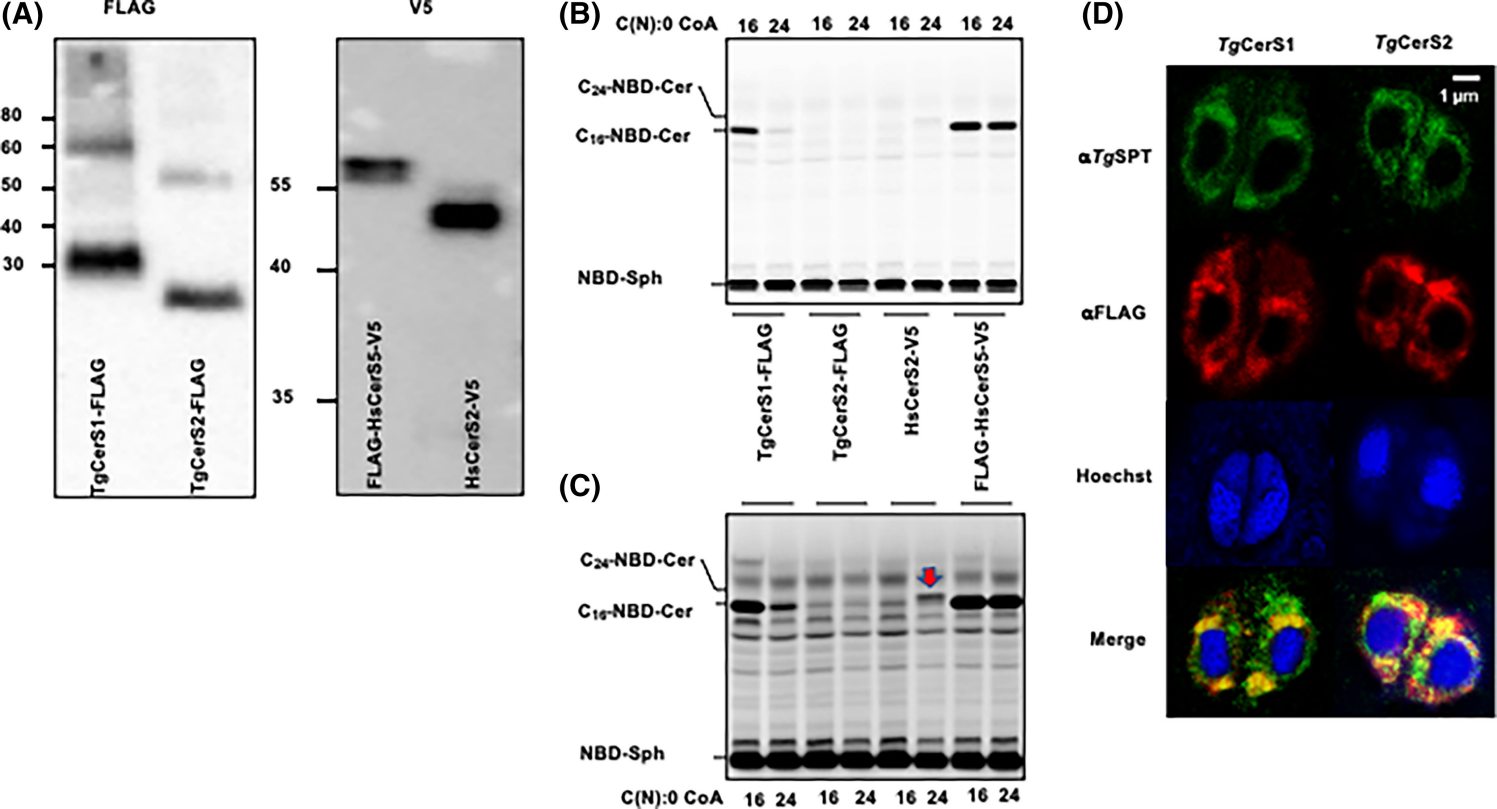
Cell‐free expression and biochemical analyses of *Tg*Cer1 and *Tg*Cer2. (A) Western blot of expressed FLAG‐tagged *Tg*Cer1 and *Tg*Cer2and V5‐tagged *Hs*CerS5 and *Hs*CerS2 probed with the primary antibodies indicated. (B) TLC separation of ceramide synthase reaction products in solvent phase CHCl_3_:MeOH:H_2_O (80:12:1 v:v:v), *Hs*CerS5 and *Hs*CerS2 are controls and provide markers for sphinganine (NBD‐Sph) and ceramide (C_16_‐NBD‐Cer and C_24_‐NBD‐Cer, respectively). Acyl‐CoA substrate for each reaction shown along top of the plate. The formation of C_16_‐NBD‐Cer by CerS5 and *Tg*CerS1 is only partially dependent on 16:0 CoA addition, presumably because the reaction mixture contains some wheat germ‐derived CoA. (C) Longer exposure of B, red arrow indicates C_24_‐NBD‐Cer produced through action of *Hs*CerS2. The relative low activity of *Hs*CerS2 is consistent with our laboratory experience and maybe due to a lack of phosphorylation^51^ or low in vitro bio‐availability of the long chain CoA. The acyl‐CoA substrate for each reaction shown along bottom of the plate. (D) Airyscan microscopy showing co‐localization of ER marker *Tg*SPT1 (in green; stained with polyclonal rat anti‐*Tg*SPT1)^20^ with *Tg*CerS1_FLAG and *Tg*CerS2_FLAG (in red; stained with monoclonal mouse anti‐FLAG). Blue is Hoechst‐stained DNA. Yellow is overlap of green (*Tg*SPT1) and red (*Tg*CerS1/2).

The in vitro study of integral membrane proteins is generally challenging. By utilizing a wheat germ‐based cell‐free membrane protein expression system, both *Tg*CerS1 and *Tg*CerS2 were expressed as FLAG‐tagged proteins in in vitro proteoliposomes. Western blot analyses utilizing anti‐FLAG rabbit monoclonal antibodies demonstrated that both proteins are produced (Figure 3A). Subsequently, biochemical analyses of the CerS activity within these proteoliposomes was undertaken utilizing fluorescent NBD‐sphinganine (NBD‐Sph) and acyl‐CoA (C16:0 or C24:0). Lipidomic analyses have indicated that the primary ceramide species in *Toxoplasma* are C‐16 and C‐18.^3^, ^8^ In line with this observation, *Tg*CerS1 demonstrated a robust CerS activity with NBD‐Sph and C16:0 acyl‐CoA as substrates. No activity was observed toward NBD‐Sph and C24:0 acyl‐CoA (Figure 3B,C). In sharp contrast, *Tg*CerS2 showed no activity to NBD‐Sph combined with either of the CoA substrates (Figure 3B,C), in keeping with the absence of the canonical residues in the Lag1 motif (Figure 2A,B). The human orthologues *Hs*CerS2 and *Hs*CerS5 were analyzed in the same manner and used as positive controls for CerS activity with C24:0 and C16:0 acyl‐CoA respectively (Figure 3B,C).

Like the active *Tg*CerS1, and other eukaryotic orthologues, the “pseudo” ceramide synthase *Tg*CerS2 localized to the ER, as shown by co‐localization with the validated ER marker *Tg*SPT1^20^, ^27^ (Figure 3D). ER localization is canonical for CerS and provided further evidence that despite the often unusual nature of the enzymes,^20^ the geometry of the sphingolipid biosynthetic pathway is conserved with respect to that seen in other eukaryotes.

### 3.3 Assessment of the roles of *Tg*CerS1 and *Tg*CerS2 in parasite fitness

**FIGURE 4:**
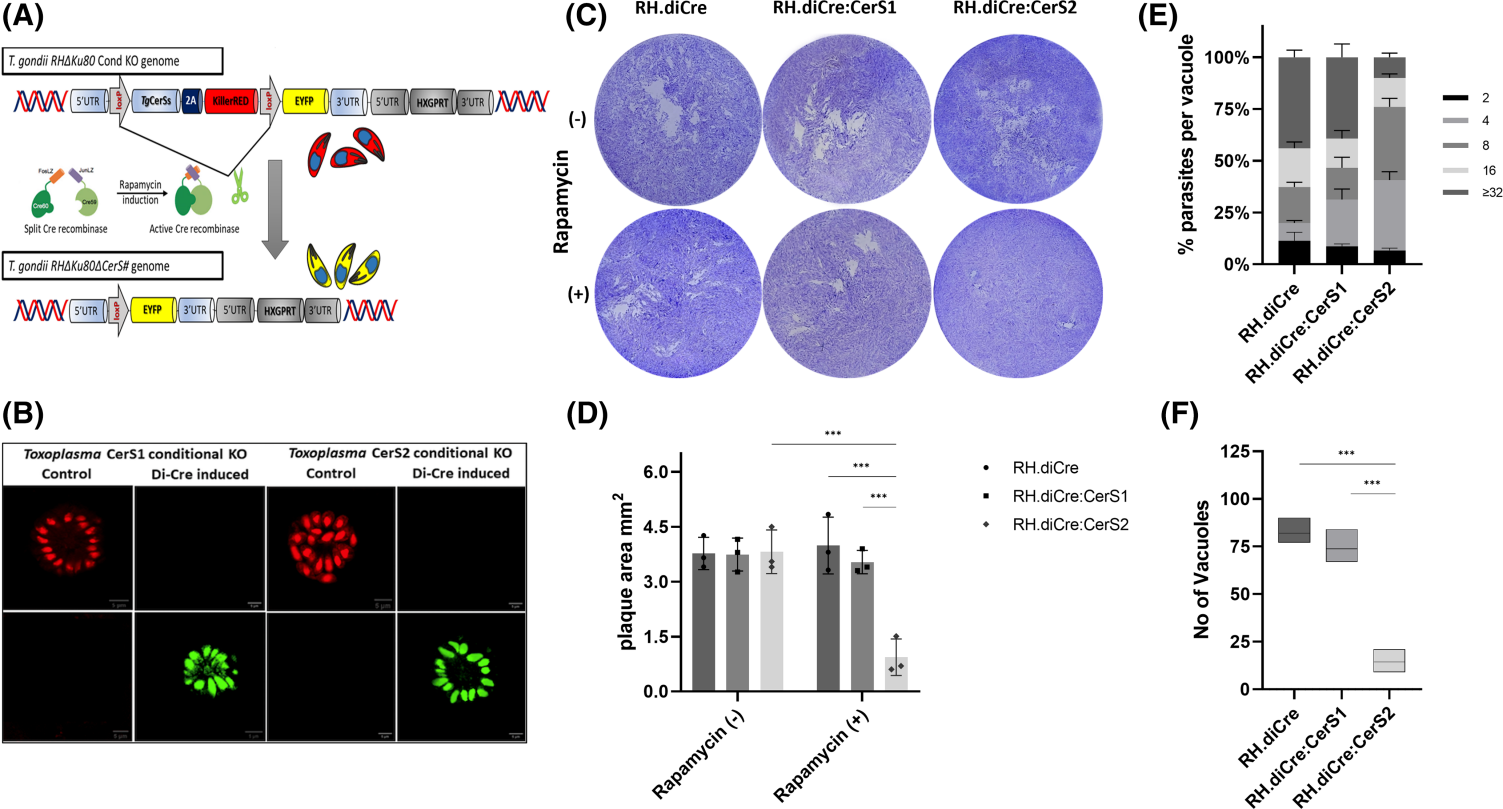
*Tg*CerS2 plays a critical role in parasite fitness in vitro. (A) Schematic illustration of the diCre mode of action and formation of *Tg*CerS1 and *Tg*CerS2 conditional knockouts (KOs). (B) Fluorescent identification of successful *Tg*CerS1 and *Tg*CerS2 deletion after 100 nM rapamycin induction. Confocal images were captured 72 h post‐infection, scale bars 5 μM. (C) Plaque assay of *Toxoplasma* (100 parasites per well in 24‐well plate) parental and *Tg*CerS1 (RH.diCre:CerS1) and *Tg*CerS2 (RH.diCre:CerS2) knockouts, uninduced or rapamycin induced (4 h), in HHF cells 12 days post‐infection. Plaques are distinguishable as clear areas on the background of a Crystal Violet stained HHF monolayer, representative images shown. (D) Area of plaque sizes in cm^2^. (E) Comparison of the intracellular replication rates of the parental and the RH.diCre:CerS1 and RH.diCre:CerS2 KOs 12 days post‐infection as determined by the distribution of the number (2, 4, 8,16, ≥32) of *Toxoplasma* parasites per parasitophorous vacuole (PV); (F) and by the number of PVs in the infected cell population (microscopic field of view). At least 150 vacuoles were analyzed for each line. PVs and intracellular parasites were analyzed from 3 slides (1.54 cm^2^) for each line, in each experiment. Values are expressed as mean ± SD of three independent experiments. *P*‐value significance thresholds were set at: *p < .05, ***p < .01, ***p < .001.

CRISPR/Cas9‐directed knockout allowed isolation of viable, but disabled, clones lacking the *Tg*CerS1 locus (Figure S4). However, equivalent *Tg*CerS2 knockout clones could not be isolated. Therefore, we developed an innovative version of the conditional diCre‐System^52^ in which the successful excision of loxP‐flanked DNA sequences is shown by a red to yellow fluorescence shift in the engineered *Toxoplasma* (Figure 4A,B; and by PCR in Figure S2). Using this system, we demonstrated that catalytically inactive *Tg*CerS2 played a greater role in parasite proliferation and, subsequently, host cell lysis than the active orthologue *Tg*CerS1 (Figure 4C). More precisely, whereas the induced *Tg*CerS1 (RH.diCre:CerS1) knockout parasites showed only a slight, statistically non‐significant reduction in plaque formation, this was greatly, and statistically significantly, reduced in the induced *Tg*CerS2 (RH.diCre:CerS2) knockout, from 3.81 ± 0.59 cm^2^ to 0.93 ± 0.49 cm^2^. No significant change was noted with the parental line on rapamycin induction or with uninduced RH.diCre:CerS1/2 (Figure 4C,D and S5). It should be noted that the monolayer disruption assay (Figure S4) broadly replicated these data, but also suggested a mild fitness phenotype for *Tg*CerS1 (RH.diCre:CerS1 and stable KO ΔCerS1).

Complementary to these analyses the parasite load and replication was assessed directly by counting the parasites per vacuole and the number of vacuoles in the infected cell population. Matching the plaque assay outcome, the *Tg*CerS2 knockout (RH.diCre:CerS2) exhibited significantly fewer parasites per vacuole compared with parental line, with the majority of the PVs found contain 4 to 8 *Toxoplasma* after 12 days, whereas >40% of vacuoles in the parental line contained ≥32 parasites (Figure 4E). Furthermore, the number of vacuoles in the infected cell population was also significantly lower in induced RH.diCre:CerS2 (Figure 4F). In contrast, in the same timeframe, the induced *Tg*CerS1 knockout (RH.diCre:CerS1) exhibited parasite per vacuole and vacuole per population profiles similar to parental line (Figure 4E,F). Again, it should be noted that the monolayer disruption assay (Figure S4) largely replicated these data.

Collectively these data indicated a clearly reduced parasite replication rate in the case of the induced *Tg*CerS2 deletion. This could explain the difficulty of isolating a *Tg*CerS2 knockout line using the direct CRISPR/Cas9 approach.

### 3.4 Phylogenetic analyses of the apicomplexan ceramide synthases

**FIGURE 5:**
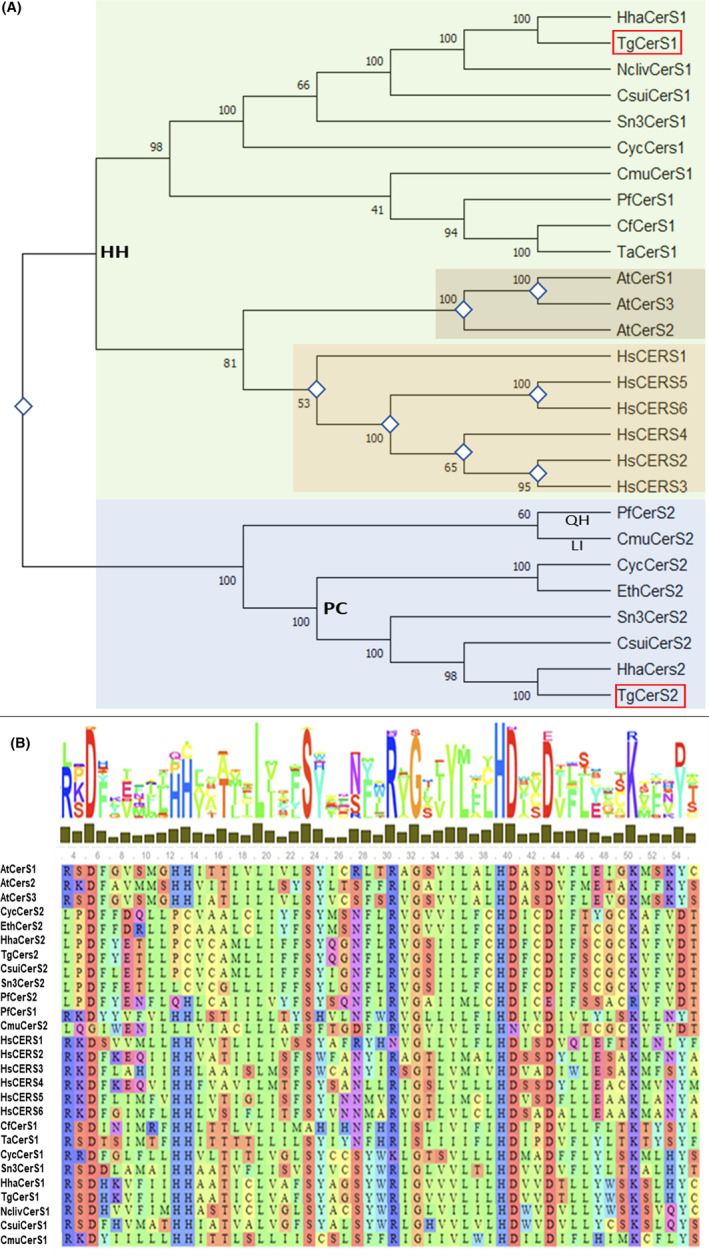
Phylogenetic analyses of the apicomplexan ceramide synthases. (A) Neighbor Joining (N‐J) tree of apicomplexan, human and *Arabidopsis thaliana* ceramide synthases analogs. Bootstrap values are based on 1000 replicates. *Toxoplasma Tg*CerS1 and *Tg*CerS2 are highlighted in red boxes. The canonical and essential ceramide synthase di‐histidine residues (HH) are highlighted in the CerS1 group, while proline‐cysteine (highlighted ‐ PC) residues are commonly found in the same position in the CerS2 cluster, with the exception of glutamine–histidine (highlighted—QH) in *Plasmodium* and leucine‐isoleucine (highlighted—LI) in *Cryptosporidium muris*. Gene duplication assessment based on N‐J tree analysis and represented by rhombuses. The distinct clades containing *Tg*CerS1 and *Tg*CerS2 are highlighted with green and purple respectively. (B) MAFFT Lag1 motif alignment of *Tg*CerS1, *Tg*CerS2 and orthologues from the Apicomplexa, humans and plants. Consensus is highlighted above. Hydrophobic amino acids are depicted in green, aromatic amino acids in cyan, aliphatic amino acids in red and orange, and large polar acids in purple and blue.^42^

Further analyses revealed that *Tg*CerS1 and *Tg*CerS2 have orthologues in the Apicomplexa. Two in *Cryptosporidium muris*, *Cystoisospora suis, Cyclospora cayetanensis, Hammodia hammondi, Plasmodium falciparum*, and *Sarcocystis neurona*, but only one copy in *Cytauxzoon felis, Eimeria tenella, Neospora canium*, and *Theileria annulata*. A region of the CerS amino acid sequences encompassing the Lag1p domain, including apicomplexan, human and *Arabidopsis thaliana*, were aligned (Figure S6) and subjected to phylogenetic analyses, including neighbor joining (Figure 5A), maximum parsimony and minimum evolution (Figure S7). In all cases, the sequences separated into two distinct and distant clades. The first is characterized by the conserved double histidine residues (H217 H218 in *Tg*CerS1) and represents the majority of the ceramide synthases, including the subgroups of *Hs*CerS1‐6, *At*CerS1‐3 and the apicomplexan orthologues of the functional ceramide synthase, *Tg*CerS1. The second clade contains the “pseudo” ceramide synthase, *Tg*CerS2, and its apicomplexan orthologues. All of these possess only one (Q209 H210, e.g., *Plasmodium* spp.) or no histidines (P178 C179, e.g. *Tg*CerS2) in place of the pair found in the other clade (Figure 5B). Furthermore, the R to L and Y to D changes found in the Lag1 domain of *Tg*CerS2 (Figure 2; at positions 169 and 220, respectively) are conserved in all members of this clade. Coupled with the evidence presented above showing that *Tg*CerS2 is not catalytical active in vitro, although still ER localized, this strongly suggested that the genes encoding this apicomplexan unique “pseudo” ceramide synthase diverged early in evolution, possibly as a result of gene duplication, where one copy of the gene maintained the original function and the other diverged to a new role. The majority of species that possess a single CerS maintain an orthologue of the functional ceramide synthase *Tg*CerS1. If an early gene duplication event led to the CerS2 orthologues, this indicated that the second copy was lost during evolution. However, surprisingly *E. tenella*, a coccidian apicomplexan like *Toxoplasma*, only maintains an orthologue of the “pseudo” ceramide synthase, *Tg*CerS2, indicating that this parasite is unable to synthesize ceramide *de novo*.

## 4 DISCUSSION

Due to the essentiality of sphingolipids,^22^ ceramide synthases (CerS) have been investigated as potential targets for therapeutic intervention in human disorders^53^, ^54^ and microbial infections.^55^ However, functional analyses to define druggability have been lacking in the Apicomplexa. To address this deficit in this study, we isolated, analyzed, and functionally defined ceramide synthase (CerS) in the model apicomplexan *Toxoplasma gondii*.

Two CerS orthologues, which we named *Tg*CerS1 and *Tg*CerS2, were identified in the *Toxoplasma* genome using bioinformatic approaches. Previously, genome‐wide, analyses had identified both of these gene products as constitutively expressed throughout the lifecycle^49^ and of some importance for parasite fitness.^48^ Here, we define the function, localization and evolutionary origin of these unusual enzymes in *Toxoplasma*. *Tg*CerS1 was found to be a fully functional CerS, with preferential use of C16:0 acyl‐CoA consistent with previous lipidomic analyses.^3^, ^8^ Surprisingly, *Tg*CerS2 was catalytically inactive as a CerS in vitro (Figure 3) and contained non‐conservative substitutions in the canonical residues defining the putative active site Lag1 motif. Utilizing the recently released AlphaFold2/RosettaFold artificial intelligence programs^43^, ^56^ we established that the structure of both orthologues was canonical, seven transmembrane domains with the substituted residues found in same arrangement in both *Tg*CerS1 and *Tg*CerS2 (Figures 2 and S3). With both programs, multiple predicted structures produced the transmembrane regions consistently and with high confidence levels. In both enzymes, the Lag1 motif is situated in the center of the trans‐membrane region forming a hydrophobic pocket perfectly suited to accommodate the lipid substrates, with the putative active histidines located close together in *Tg*CerS1. Importantly, the changes in the *Tg*CerS sequence, active *Tg*CerS1 to inactive *Tg*CerS2, lead to no significant structural change in the putative active site indicating that *Tg*CerS2 may still bind the same substrates when the catalytic activity as a ceramide synthase is lost. However, the soluble loop regions are predicted with low confidence and show limited consistent three‐dimensional structure. The “Hox‐like” domain which was predicted in the human CerSs^57^, ^58^ following the first transmembrane helix is not present in either *Tg*CerS1 or 2. This is not surprising as the enzyme is highly unlikely to have any DNA‐binding functions. Notably, there are large differences between the AlphaFold2 and RosettaFold predictions of the *Tg*CerS1 and *Tg*CerS2 cytosolic face structures, in explanation the C‐terminus could represent different conformations—such as the opening and closing of loops over the active side. The models for the luminal side, the N‐terminus, show even lower confidence levels and are not even consistent with respect to secondary structure. Only experimental structure determination by X‐ray crystallography or cryo‐Electron Microscopy will be able to resolve the discrepancies between the models and allow the further elucidation of function.

Using an immunofluorescent approach both proteins were identified as localized in the *Toxoplasma* ER, the canonical localization of CerS (Figure 3). Together, these data indicated that *Tg*CerS2, while non‐catalytic, had maintained structure, localization, and, presumably, functionality through evolution. Using an inducible knockout approach, in agreement with data from the genome‐wide screen (Table 1),^48^ deletion of *Tg*CerS1 exhibited only a modest fitness phenotype in an in vitro tachyzoite infection assay (Figures 4 and S4). However, in contrast, loss of the non‐catalytically active *Tg*CerS2 demonstrated a statistically significant effect (Figures 4 and S4). This deviated from the data generated during the genome‐wide screen,^48^ which suggested that loss of this protein had a minimal effect (Table 1). However, in agreement with the inducible KO phenotype, and unlike with *Tg*CerS1, in an orthologous approach conventional CRISPR/Cas9 KO of *Tg*CerS2 was unachievable in our study. Similarly, in functional studies of *Tg*SPT1 and 2,^27^ the former exhibited a major defect in an equivalent in vitro assay despite the genomic screen data suggesting this protein was non‐essential for fitness (Fitness Score −0.62^48^). The reasons for these deviations are unclear; however both this study, and the previous *Tg*SPT1 analyses,^27^ indicate that re‐examination of gene and protein essentiality in *Toxoplasma* is warranted.

From where did this “pseudo” enzyme arise? Further bioinformatic analyses demonstrated that orthologues of *Tg*CerS1 could be identified in almost all members of the Apicomplexa, and *Tg*CerS2 orthologues were found in a large subset. Phylogenetic analyses (Figures 5A and S7) clearly demonstrated that the predicted ceramide synthases divide into two clades, one containing the *Tg*CerS1 orthologues and including the *Arabidopsis* and human enzymes, the other clustered the *Tg*CerS2 “pseudo” enzyme orthologues from the Apicomplexa. The fact that all the *Tg*CerS2 orthologues maintain the R‐L and Y‐D changes (positions 169 and 220 in *Tg*CerS2) and the loss of canonical HH (positions 217 and 218 in *Tg*CerS2), to PC in all aside from *Plasmodium* spp. (QH) and *C. muris* (LI), supported early divergence and the maintenance of key functionality (Figure 5). However, despite this putative selective pressure, *Cytauxzoon felis, Theileria annulata*, and *Neospora canium* encode only a *Tg*CerS1 orthologue (Figure 5B). While *C. felis* and *T. annulata* are of the same family, Theileriidae, *N. canium* is in the Sarocystidae. *Sarcocystis neurona, Cystoisospora suis* and *Hammodia hammondi* are also in this latter family and maintain orthologues of both *Tg*CerS1 and *Tg*CerS2 (*T. gondii* is also in the Sarocystidae). This suggested that the loss of the *Tg*CerS2 orthologue occurred later in evolution due to unknown environmental stresses and evolutionary pressures.

In contrast, and interestingly, *E. tenella* maintain a *Tg*CerS2 orthologue but appears to have lost the active ceramide synthase which indicated that this parasite lacks the ability to synthesize ceramide *de novo* (Figure 5). This further supported the importance of the non‐catalytic ceramide synthase, a hypothesis reinforced by the same gene arrangement being found other *Eimeria* species (see Materials and Methods). However, uniquely in the *Eimeria* species, *E. falciformisi* maintains the functional *Tg*CerS1 and “pseudo” *Tg*CerS2 orthologue arrangement (EfaB_MINUS_15758.g1433 and EfaB_PLUS_2387.g283, respectively). Interestingly, *E. falciformisi* is the only *Eimeria* species in which the sphingolipid content has been extensively analyzed, with a lipidomic approach indicating that *de novo* ceramide biosynthesis occurs.^9^ Like *E. falciformisi*, another member of the family Eimeridae, *Cyclospora cayetanensis*, also encoded ortholgues for both proteins. Together, these data indicated that loss of orthologues of either protein (*Tg*CerS1 or *Tg*CerS2) is a relatively recent event in evolution and that at least one must be maintained. Apart from in most *Eimeria* species, the maintained orthologue appears to be a catalytical active ceramide synthase.

However, the maintenance of apicomplexan *Tg*CerS2 orthologues, either alongside a functional enzyme or, in the *Eimeria* species, alone is mysterious given its apparent lack of catalytic activity. Hypothetically, following gene duplication, one copy could become non‐functional due to degenerative mutations. However, alongside the situation in *Eimeria* species and the key role in *Toxoplasma* fitness reported here (Figure 4), the conservation of amino acids in the Lag1 motif and of the tertiary structure (Figure 2) in these orthologues make this unlikely. Alternatively, a duplicated copy could acquire a new function that is favorable for the organism. The maintenance of *Tg*CerS2 orthologues, and its seemingly non‐catalytic nature, across the Apicomplexa support this hypothesis. However, the true function of the *Tg*CerS2 orthologues remains unknown. Given the proposed dimerization of human CerS2 and CerS5 to modulate activity^59^ we investigated the possibility of *Tg*CerS1 and *Tg*CerS2 dimerization using the predicted structures (Figure 2) and those similarly generated for human CerS2 and CerS5. However, no evidence for the formation of dimers was provided for either pair (data not shown), although apparent *Tg*CerS1 and *Tg*CerS2 homodimers were seen by Western blotting (Figure 3A). In addition, the conservation of *Tg*CerS2 alone in the *Eimeria* spp. renders this explanation unlikely. An alternative hypothesis would be that the non‐catalytic isoforms act as ceramide binders and regulate the availability of this highly bioactive lipid. This is a particularly attractive explanation for the *Eimeria* species which presumably would have to scavenge and regulate host ceramide. However, while functional analyses of these essential proteins in an obligate intracellular pathogen of a host encoding the same activity are highly challenging, future detailed analyses to fully understand the roles of the apicomplexan “pseudo” enzyme could make use of the stable *Tg*CerS1 knockout reported here (KO ΔCerS1; Figure S4). This mutant has a limited fitness deficit (Figure S4), although was recently shown by us to have a distinctive morphological phenotype.^60^ Hypothetically, this transgenic line is devoid of *de novo* ceramide synthase activity and therefore reliant of the acquisition of host lipid. In such a background, the host requirement for host ceramide synthesis could be probed using either broad spectrum or isoform specific host ceramide synthase inhibitors^54^, ^61^, ^62^ and low serum media.^8^

However, the identification and analyses described here detail a system unique to the Apicomplexa, a phylum of parasitic protozoa. Given the importance to parasite proliferation, coupled with the non‐mammalian nature of ceramide synthesis and regulation, this could represent a potential point for therapeutic intervention for a range of diseases from malaria to toxoplasmosis.

## AUTHOR CONTRIBUTIONS

*Conceptualization and project administration*: Paul W. Denny. *Funding acquisition and supervision*: Paul W. Denny and Ehmke Pohl. *Investigation*: Zisis Koutsogiannis, John G. Mina, and Christin A. Albus. *Methodology*: John G. Mina, Christin A. Albus, Ehmke Pohl, and Matthijs A. Kol. *Visualization*: Paul W. Denny, Zisis Koutsogiannis, and John G. Mina. *Writing—original draft*: Paul W. Denny, Zisis Koutsogiannis, John G. Mina, and Christin A. Albus. *Writing—review and editing*: Paul W. Denny, Zisis Koutsogiannis, John G. Mina, Matthijs A. Kol, Joost C. M. Holthuis, and Ehmke Pohl.

## DISCLOSURES

The authors declare no conflicts of interest.

## Supporting information


Data S1

## Data Availability

The data that support the findings of this study are available in the supplementary material of this article.
