# Supplementary material for: Toxoplasma ceramide synthases: Gene duplication, functional divergence, and roles in parasite fitness
Source: FASEB J. 2023 Oct 5;37(11):e23229. doi: 10.1096/fj.202201603RRR (PMC10946778; doi:10.1096/fj.202201603RRR)
Supplement: Supplementary file 1 — Data S1 [file FSB2-37-0-s001.pdf]

Supporting Information

Fig. S1. Map of ToxoXpress which was designed, created and validated in-house for either transient expression or integration into the UPRT locus (via UPRT 5'UTR [in yellow] and UPRT 3'UTR [in cyan]) and selection using 5-fluoro-2'deoxyuridine. In both cases expression is driven by the GRA1 promotor (GRA1 5'UTR [in green]).

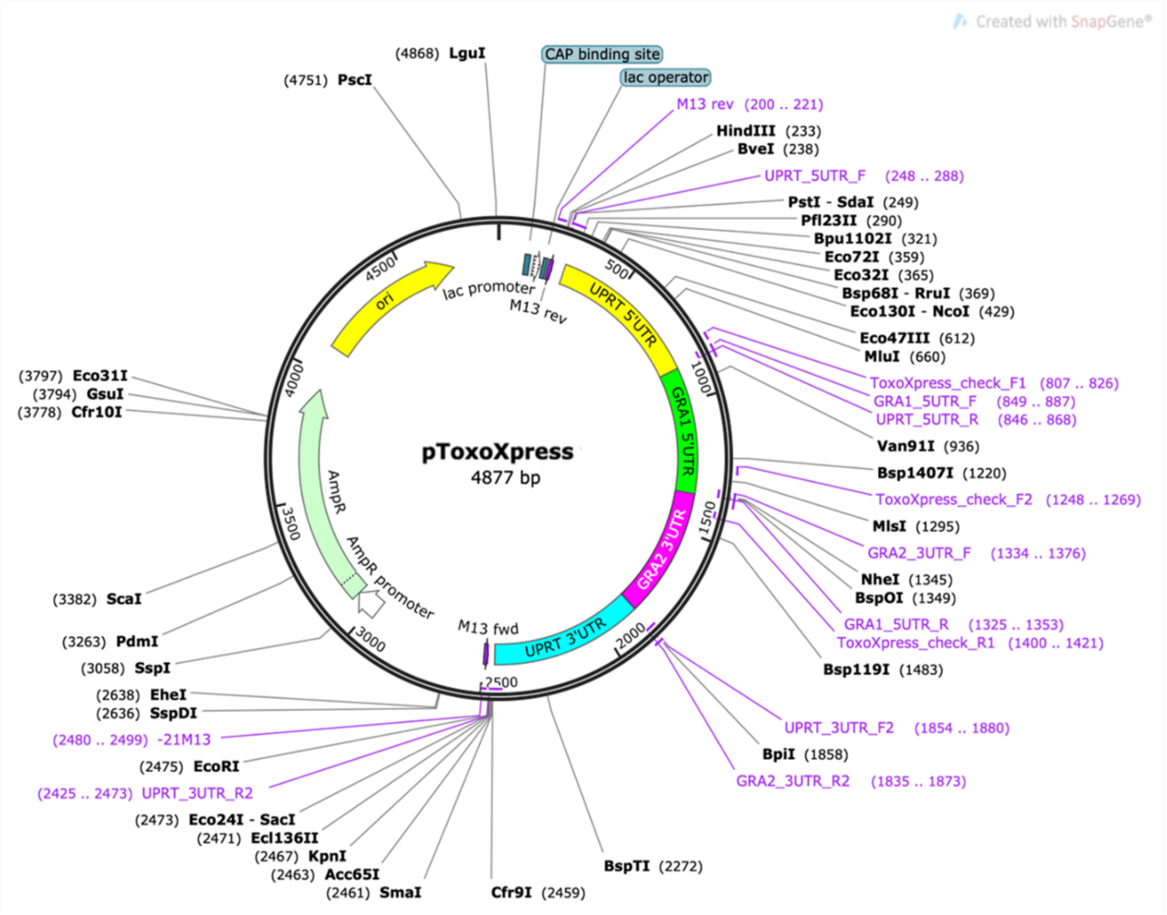

Fig. S2. Molecular identification of RH.diCre:CerS1 and RH.diCre:CerS2 transgenic clones by genetic mapping (A; clones a, b and c) and validation of rapamycin induced KO (B; left uninduced [clones a and b], right 4 hours post-induction [clone a]). Schematic illustrating approach and primers corresponding to supplementary tables S8 and S9 (C).

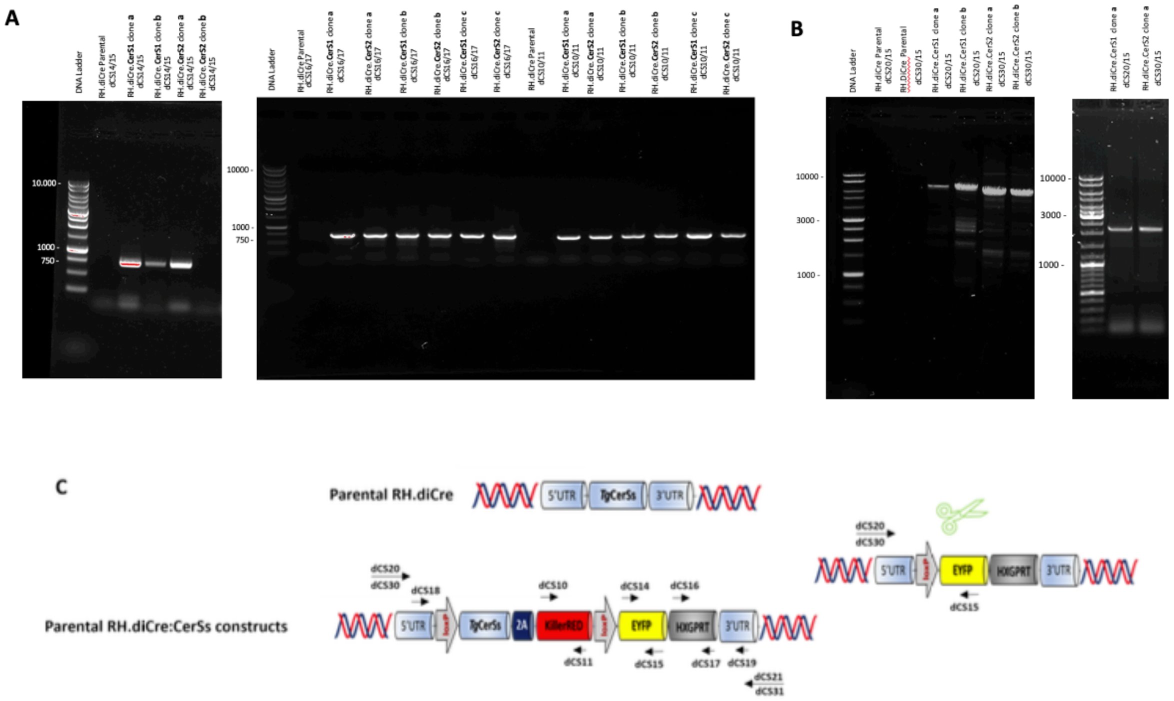

Fig. S3. Ribbon diagram of I. *Tg*CerS1 and II. *Tg*CerS2 as predicted by (A) AlphaFold2 (1) and (B) Rosetta fold (2). Transmembrane domains are shown in different shades of cyan from H1 - light to H7 dark. Arginine (R208), double histidine (H217 H218 and tyrosine (Y259) residues in *Tg*CerS1, as well as leucine (L168), proline (P178), cysteine (C179) and aspartic acid (D220) residues in *Tg*CerS2, are highlighted in magenta and shown in stick formation. Putative extracellular helices shown in red, strands in yellow and loops in green. C-termini point up towards cytosol.

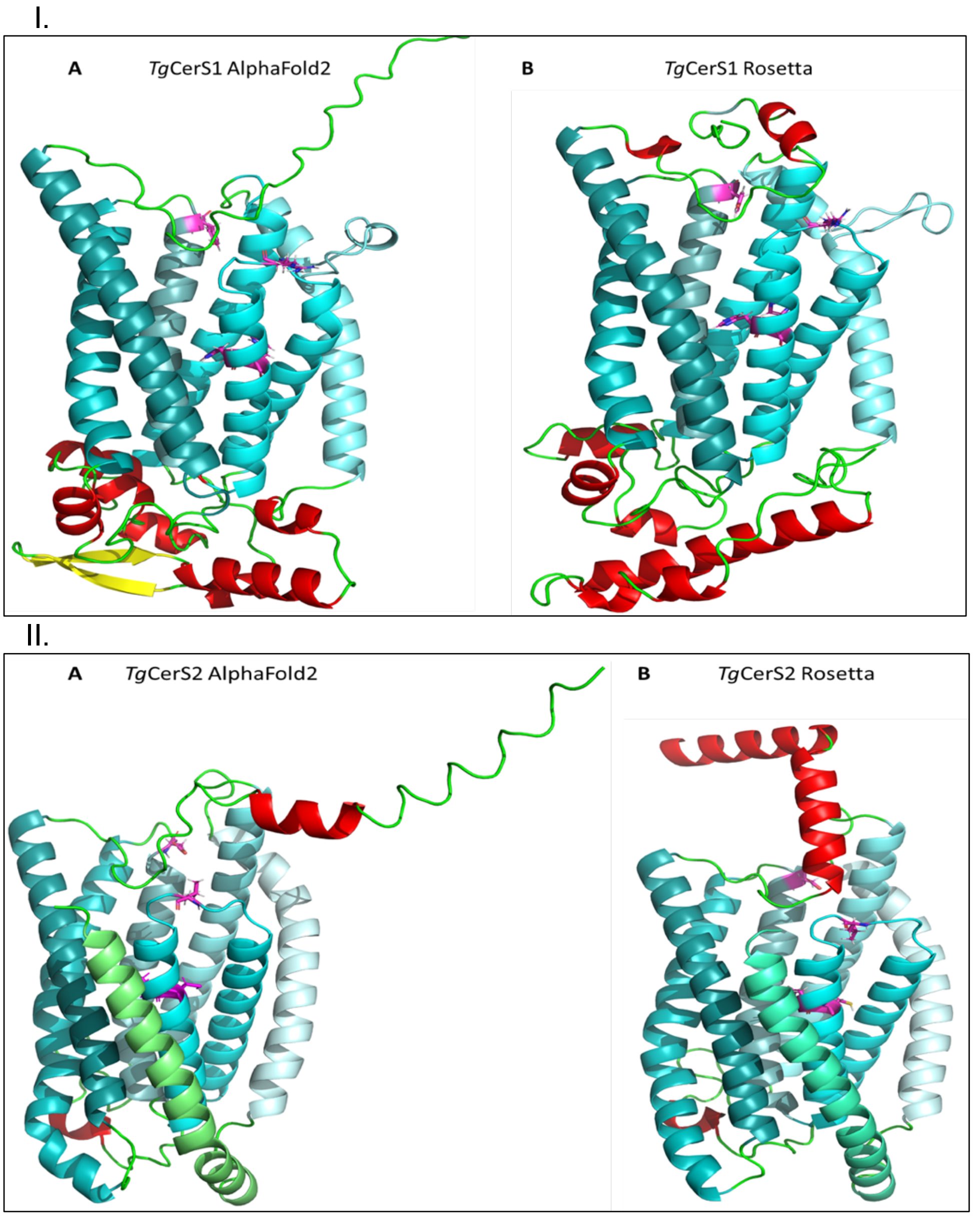

Fig. S4. Monolayer disruption fitness assay of *Toxoplasma* parental (RH.diCre); rapamycin-induced *Tg*CerS1 and *Tg*CerS2 knockouts (RH.diCre:CerS1 and RH.diCre:CerS2; 4 hour induction); and non-conditional Δ*Tg*CerS1, at 48-, 72- and 96 hours post infection with 10^5 parasites per well (A); Area of host cell lysis in cm^2, values are expressed as mean $cm^2 \pm$ SD of three independent experiments analysed with ImageJ (B); Comparison of the intracellular replication rates of the parental and RH.diCre:CerS1 and RH.diCre:CerS2 72 hours post infection, as determined by the distribution of the number of *Toxoplasma* parasites per parasitophorous vacuole (PV; 2, 4, 8,16, ≥32) (C); and by the number of PVs in the infected cell population (microscopic field of view; D). At least 150 vacuoles were analysed for each strain. PVs and intracellular parasites were analysed from 3 slides (4 cm^2) for each strain in each experiment.

P value significance thresholds were set at: *p<0.05, **p<0.01, *** p<0.001.

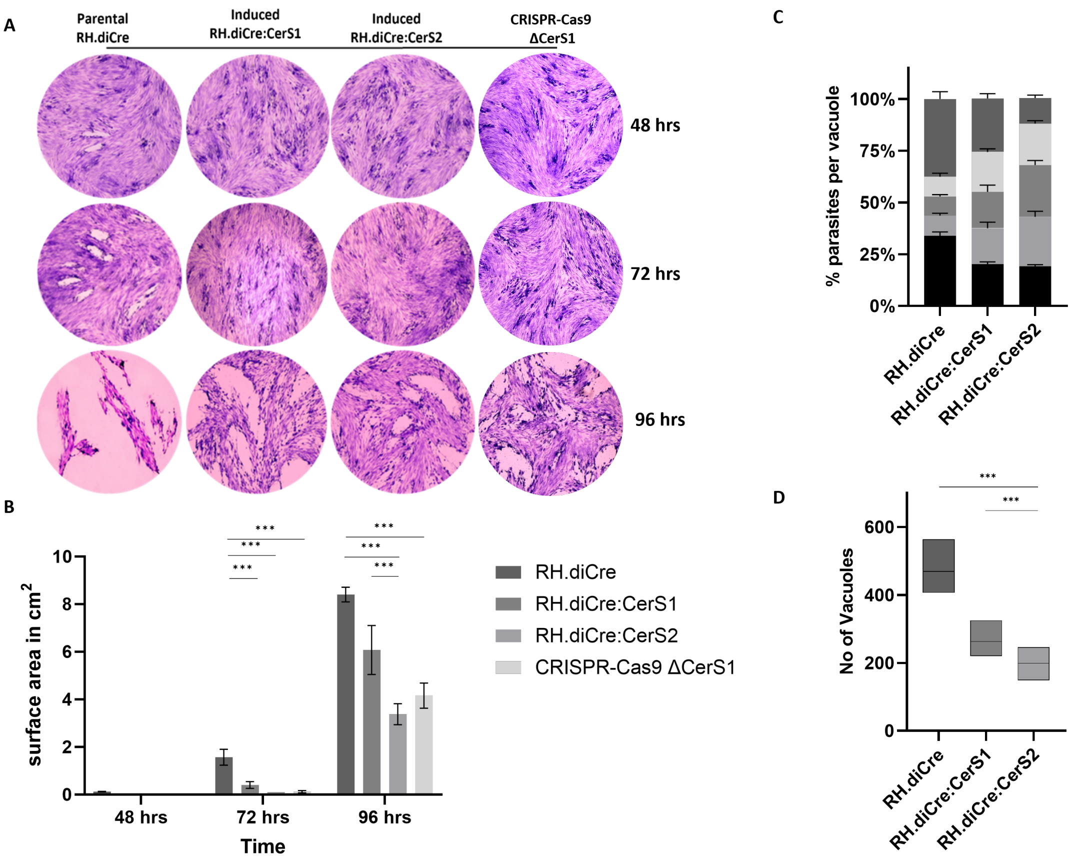

A
Parental RH.diCre
Induced RH.diCre:CerS1
Induced RH.diCre:CerS2
CRISPR-Cas9 ΔCerS1
48 hrs
72 hrs
96 hrs
B
surface area in cm2
0
2
4
6
8
10
48 hrs
72 hrs
96 hrs
Time
***
RH.diCre
RH.diCre:CerS1
RH.diCre:CerS2
CRISPR-Cas9 ΔCerS1
C
% parasites per vacuole
0%
25%
50%
75%
100%
RH.diCre
RH.diCre:CerS1
RH.diCre:CerS2
2
4
8
16
32
D
No of Vacuoles
0
200
400
600
***
RH.diCre
RH.diCre:CerS1
RH.diCre:CerS2

Fig. S5. Monolayer disruption fitness assay of parental RH.diCre (positive control), uninduced RH.diCre:CerS1 and RH.diCre:CerS2, with uninfected HFFs as a negative control at 48-, 72- and 96 hours post infection with 10^5 parasites per well.

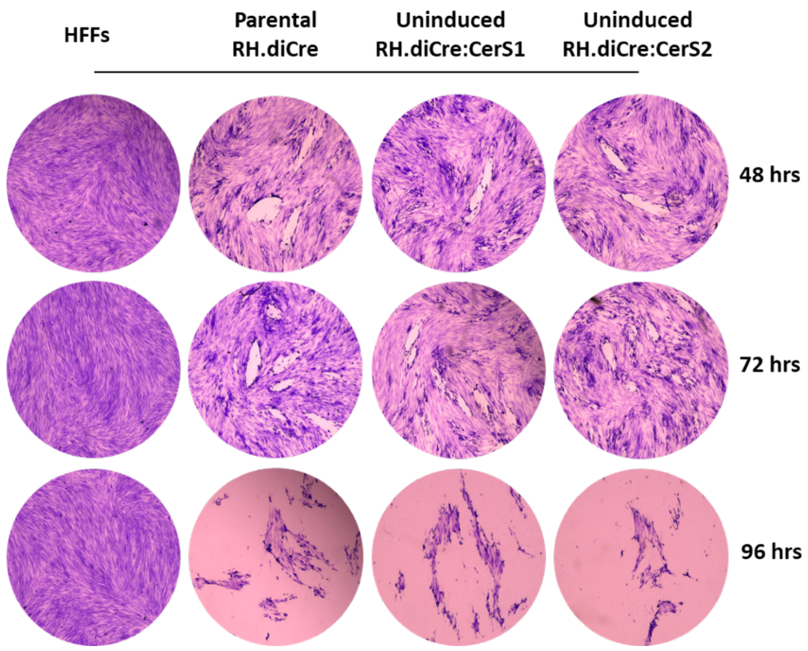

Fig. S6. Sequence alignment used for phylogenetic analyses (ClustalW). Region includes the lag1p motif and is equivalent to residues 197-332 of *Tg*CerS1.

```
CmuCerS2        FSLFCLLNEEPLQGIWENILLIVIACLLLAFSFTGDFIRVGVIVLFLHNVCDILTCGCKV
PfCerS2         TSVYSLLISEKLPDFYENFLQHLCAIILVYFSYSQNFIRVGAIIMLCHDICEIFSSACRV
CycCerS2        ASLVCILKSPWLPDFFDQLLPCVAALCLIYFSYMSNFLRVGVVILFCHDICDIFTYGCKA
EthCerS2        ASLIYILKSPWLPDFFDRLLPCAAALCLIYFSYMSNFLRVGVVILFCHDICDIFTCGCKA
HhaCerS2        ACFISLLLSPKLPDFYETLLPCVCAMLLIFFSYQGNFLRVGSIILFCHDFCDIFSCGCKV
TgCerS2         ACFISLLLSPKLPDFYETLLPCVCAMLLIFFSYQGNFLRVGSIILFCHDFCDIFSCGCKV
CsuiCerS2       SCFILLLLSPKLPDFLETLLPCVCAMLLIFFSYIGNFLRVGSIILFCHDFCDIFSCGCKV
Sn3CerS2        SCFLSLMLAPKLPDFFETLLLCVCGLLLIFFSYLGNFLRVGSIILFCHDFCDIFSCGCKV
CmuCerS1        SCLIIILNFETIRKDYIILLLHHITTLSLLIISCSLSFFRIGIIVLWIHDILDIFLHIMKC
CfCerS1         SCLLYVNCETRRSDINIMRFHHLTTLVLIIMAHIHNFHRISLIVIFIHDIPDVLLFLTKT
TaCerS1         SCLIFIRWETRRSDTSIMTFHHITTTTLLILSYIYNFHRISIIIIFLHDIPDVFLYLTKT
PfCerS1         SCLFYLNYEIRRKDYYVFVLHHLSTIILLTYSHVLNFWRVGLLILFIHDIVDIVLYLSKL
CycCerS1        TCLVFMNLETRRRDFGLFLLHHVLTITLVGLSYCCSYWKLGTSVLLLHDMADFFLYLSKM
Sn3CerS1        SSVCYLFADVRRSDDLAMAIHHAATVFLISVSYVCSYWRLGLVVLTLHDVVDVFLYLTKA
CsuiCerS1       SCLVFLRFETRRSDFHVMATHHIATVALVGFSYALSYWRLGHVVLVLHDWVDVLLYCSKS
HhaCerS1        SCSLFLRIETRRSDHKVFIIHHAATICLVAFSYAGSYWRIGVVVLILHDVVDTLLYWSKS
TgCerS1         SCSLFLRIETRRSDHKVFIIHHAATICLVAFSYAGSYWRIGVVVLILHDVVDTLLYWSKS
NclivCerS1      SCAVFLRIETRRSDHVVFIMHHASTVCLVGLSYACSYWRIGLVILILHDWVDVLLYWSKS
AtCerS2         GVAALLAWETRRKDFAVMMSHHVITIILLSYSYLTSFFRIGAIILALHDASDVFMETAKI
AtCers1         SIFALVFWETRRSDFGVSMGHHITTLVLIVLSYICRLTRAGSVILALHDASDVFLEIGKM
AtCerS3         SIFALVFWETRRSDFGVSMGHHIATLILIVLSYVCSFSRVGSVVLALHDASDVFLEVGKM
HsCERS1         SIYATLYMDTWRKDSVVMLLHHVVTLILIVSSYAFRYHNVGILVLFLHDISDVQLEFTKL
HsCERS5         SLMFSQFTDIKRKDFLIMFVHHLVTIGLISFSYINNMVRVGTLIMCLHDVSDFLLEAAKL
HsCERS6         SLMFSQFTDIKRKDFGIMFLHHLVSIFLITFSYVNNMARVGTLVLCLHDSADALLEAAKM
HsCERS4         SLLIRLPFDVKRKDFKEQVIHHFVAVILMTFSYSANLLRIGSLVLLLHDSSDYLLEACKM
HsCERS2         SLLFSIASDVKRKDFKEQIIHHVATIILISFSWFANYIRAGTLIMALHDSSDYLLESAKM
HsCERS3         SLLFRLGFDVKRKDFLAHIIHHLAAISLMSFSWCANYIRSGTLVMIVHDVADIWLESAKM
                             .             *:  :      . .  ::  *:  :      :

CmuCerS2        FVDTK---------WQAITLGLFGILLAAWA--YLRIYCFSRIVLYPVYGQ-------I-
PfCerS2         FVDTR---------YKFITVTSFCILFTSWG--FLRLYIFVKRCILPIHRN-------FD
CycCerS2        FVDTP---------YHKVTIGLFMLLTTCWF--YFRLYTFPAAALFPIFKA-------IK
EthCerS2        FVDTP---------YHKVTIGLFVLLTVCWF--YFRLYTFPSAALFPIYKA-------IK
HhaCerS2        FVDTR---------HKVVTFFLFACLVVSWG--YLRLFAFPVAALFPIFKN-------VK
TgCerS2         FVDTR---------HKVVTFFLFACLVVSWG--YLRLFAFPVAALFPIFKN-------VK
CsuiCerS2       FVDTR---------HKVVTFFLFACLMISWG--YLRLYAFPAAALFPIFKN-------VK
Sn3CerS2        FVDTR---------HKLVTFCLFACLVISWG--YLRLYTFPVATLFPIFKN-------VK
CmuCerS1        FLYSKY-------AERFPTFCNFMLYSLTLIMFISRLMIYPYFCIYSI---PIIRTYTNA
CfCerS1         YSYINN-------KNETMLGSLFLLYGLSHF--ITRFVLLISYVGYPLLFKLDAFHYNGG
TaCerS1         YSYFTR-------KNEILLSLFFVTYGLSHF--IARFVLLLRYIAYPLLINFDNFEYSGG
PfCerS1         LNYTNL-------KNRIFLTFFYILFVLYYF--FFRIFLYFYYIVLPLSNTKIIRSYTDG
CycCerS1        LHYSRV--------KSGPVEIAFATFTIVFF--VARLVLYPLYCVRPCLNTALIKEFTRD
Sn3CerS1        LHYTFL--------PGKLSETAFAGLLCSYF--VARLLLFPLCCVRPVVDLRYITAVFPQ
CsuiCerS1       LQYSFI--------PPRVTDCSFVCFVISYL--VARLILFPIYCVWPTIDPTLTHRLSHG
HhaCerS1        LHYCYL--------PSIVTECFFLFFVFSYL--VARLLLFSFYCVWPSIDPSYTDLLTNG
TgCerS1         LHYCYL--------PSIVTECSFLLFVFSYL--VARLLLFPFYCVWPSIDPSYTDLLTNG
NclivCerS1      VQYCYV--------PSLVVECGFVFFVVSYL--VARLLLFPFYCVWPAIDSSYTNRLTNG
AtCerS2         FKYSE---------KEFGASVCFALFAVSWL--LLRLIYFPFWIIRATSIE----LLDYL
AtCers1         SKYCG---------AESLASISFVLFALSWV--VLRLIYYPFWILWSTSYQ--II-MTVD
AtCerS3         SKYSG---------AERIASFSFILFVLSWI--ILRLIYYPFWILWSTSYE--VVLELDK
HsCERS1         NIYFKSRGGSYHRLHALAADLGCLSFGFSWF--WFRLYWFPLKVLYATSHC---------
HsCERS5         ANYAK---------YQRLCDTLFVIFSAVFM--VTRLGIYPFWILNTTLFE---------
HsCERS6         ANYAK---------FQKMCDLLFVMFAVVFI--TTRLGIFPLWVLNTTLFE---------
HsCERS4         VNYMQ---------YQQVCDALFLIFSFVFF--YTRLVLFPTQILYTTYYE---------
HsCERS2         FNYAG---------WKNTCNNIFIVFAIVFI--ITRLVILPFWILHCTLVY---------
HsCERS3         FSYAG---------WTQTCNTLFFIFSTIFF--ISRLIVFPFWILYCTLIL---------
                                                   *:

CmuCerS2        --SLALSTRLCAYSTFLLMTLLLVNIYW
PfCerS2         IFIKYLKVETCIWLIFLLLVILLMNTYW
CycCerS2        TRPHYSETEGGSYFIFILLTLCLMNIYW
EthCerS2        TRPDHSETEGSSYFIFILLTLSLMNIYW
HhaCerS2        SMKATADGEDWGFFVCLLLTLFVMNIYW
TgCerS2         SMKATADGEDWGFFVCLLLTLFVMNIYW
CsuiCerS2       NMKATADSEDWGFFVFLLLTVFVMNIYW
Sn3CerS2        DRKLTAENEGWGFFVFLLLTLFVMNIYW
CmuCerS1        AGYHLWIIPGSVICSCLLLFLQFIHIIW
CfCerS1         TIKYLWDFPGGIICPISVLCLTIMNAYW
TaCerS1         TIRYLWDFPGGIICPISIIILTIMNAYW
PfCerS1         FISSHKDVPGGLVLLIFLWTLMAMHVYW
CycCerS1        FVDSRWHIPGGTVLPGFLVVLQVLHIYW
Sn3CerS1        KMLHCFIFPGGILLPALLVVLLVLHVYW
CsuiCerS1       RVTDHHSFPGGVALPVFLCVLVGLHIYW
HhaCerS1        RVPHRFGFPGGILLPSLLCVLVGLHLYW
TgCerS1         RVPHRFGFPGGIVLPSLLCVLVGLHVYW
NclivCerS1      RLKNRFGFPGGVLLPCLLCVLVGLHVYW
AtCerS2         DMTSAEGTLMYYSFNTMLLMLLVFHIYW
AtCers1         KEKHPNGPILYYMFNTLLYFLLVLHIFW
AtCerS3         DKHPIEGPIYYYMFNTLLYCLLVLHIYW
HsCERS1         SLRTVPDIPFYFFFNALLLLLTLMNLYW
HsCERS5         SWEIIGPYASWWLLNGLLLTLQLLHVIW
HsCERS6         SWEIVGPYPSWWVFNLLLLLVQGLNCFW
HsCERS4         SISNRGPFFGYYFFNGLLMLLQLLHVFW
HsCERS2         PLELYPAFFGYYFFNSMMGVLQLLHIFW
HsCERS3         PMYHLEPFFSYIFLNLQLMILQVLHLYW
                                 :  :  .:  *
```

Fig. S7. Phylogenetic trees of selected CerS orthologues as reconstructed by MEGA-X using UPGMA, Maximum likehood and N-J dedrograms based on Poisson and number of differences models. Bootstrap values are based on 1000 replicates. *Toxoplasma Tg*CerS1 and *Tg*CerS2 are highlighted in red boxes.

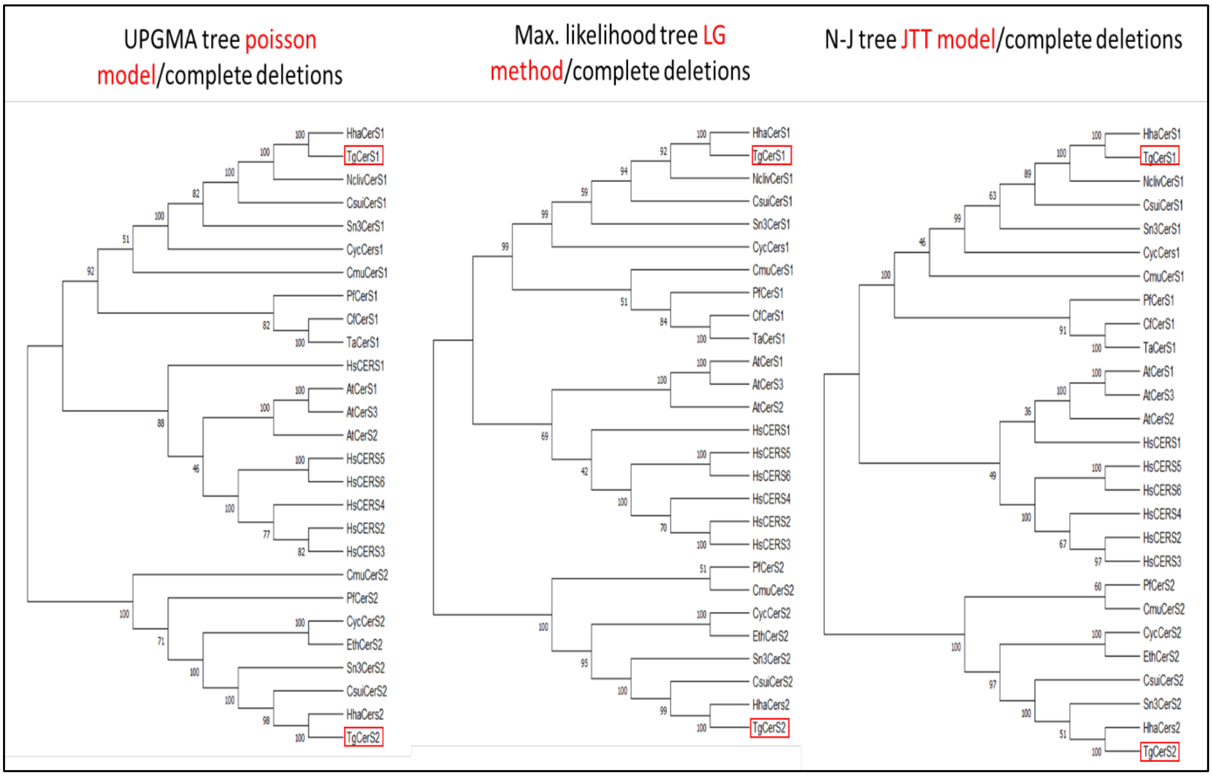

Table S1. List of primers used to create plasmids for *Tg*CerS1, *Tg*CerS2, *Hs*CerS2 and *Hs*CerS5 for cell-free expression and for site directed mutatgenesis.

Primer name	Forward sequence	Reverse sequence	Method
*Tg*CERS1.CFE.P1<br>*Tg*CERS1.CFE.P2.FLAG	CGTACGCGGGCGGCCGCATGA<br>CGCCTGTTCCCTACCAG	CAATTTATGGCGGCCGCTTACTT<br>ATCGTCGTCATCCTTGTAATCCA<br>TCCGGGTTTTGCACTTTG	In-<br>Fusion<br>cloning
*Tg*CERS2.CFE.P1<br>*Tg*CERS2.CFE.P2.FLAG	CGTACGCGGGCGGCCGCATGA<br>AGTTGGATCCGTTTTTCACCT	CAATTTATGGCGGGCTTACTTAT<br>CGTCGTCATCCTTGTAATCCATG<br>TGGCGCTTCTTGCTA	In-<br>Fusion<br>cloning
pEu.Flexi.SDM.P1<br>pEu.Flexi.SDM.P2	GCTGGTGAATGACACAGGTAC<br>C CCTATCCCTAACCCTC	GAGGGTTAGGGATAGGGGTACC<br>TGTGTCATTCACCAGC	SDM
*Hs*CERS2. CFE. P1<br>*Hs*CERS2/5.CFE.P2	GAGA CTCGAG<br>GCGATCGCGCC<br>ATGCTCCAGACCTTGTATG	GAGAGGTACC<br>TCAATGGTGATGGTGATGATG	T4
*Hs*CERS5. CFE. P1<br>*Hs*CERS2/5.CFE.P2	GAGA CTCGAG<br>GCGATCGCGCC<br>ATGGATTACAAGGATGACG	GAGAGGTACC<br>TCAATGGTGATGGTGATGATG	T4

Table S2. List of primers used to create *Tg*CerS1 and *Tg*CerS2 plasmids for subcellular localisation studies.

Primer name	Forward sequence	Reverse sequence	Method
*Tg*CerS1Xpress_F<br>*Tg*CerS1FLAGXpress_R	CCTAGCAAGATGACGCCTGT<br>TCCCTACCAGC	GTCAACACTTGCTAGTCACTTG<br>TCGTCATCGTCTTTGTAGTCGC<br>TACCTCCGCCACCCCGGGTTTT<br>GCACTTTGAATTC	RF-<br>Cloning
TgCerS2Xpress_F<br>TgCerS2FLAGXpress_R	GTCACATTTGAAGAAAGCTA<br>GCAAGATGAAGTTGGATCC<br>GTTTTTCACCTTCC	GTCAACACTTGCTAGTCACTTG<br>TCGTCATCGTCTTTGTAGTCGC<br>TACCTCCGCCACCGTGGCGCT<br>TCTTGCTATCGGA	RF-<br>Cloning

Table S3. Primers used for pTOXOXpress construction. UPRT 5' flanking region, GRA1 5'UTR, GRA2 3'UTR and UPRT 3' flanking region were amplified from RHdeltaKu80 genomic DNA and integrated into BamHI-linearised pUC19 using in-fusing cloning.

Primer name	Sequence
UPRT_5UTR_F	CAGGTCGACTCTAGAGGATCCAGCCGCTGTTTTCCAACGCA
UPRT_5UTR_R	TTTAGAAGCCCTGTGGACAGGTC
GRA1_5UTR_F	CTGTCCACAGGGCTTCTAAAGTCACCTGGCGTCTCAGCT
GRA1_5UTR_R	CTTGCTAGCTTTCTTCAAAGAACAACAGC
GRA2_3UTR_F	CTTTGAAGAAAGCTAGCAAGTGTTGACTACGACGAAAGTGATG
GRA2_3UTR_R2	GATTCCGTCAGCGGTCTGTCTTCTGCTGCCAGATGCTGC
UPRT_3UTR_F2	GACAGACCGCTGACGGAATCGCGGACT
UPRT_3UTR_R2	AGCTCGGTACCCGGGGATCCCATCCCACACCGTTAAAATTCCGTTAGAG

Table S4. List of primers used to create CRISPR/Cas-9 plasmids. sgRNAs were desighed to target *Tg*CerS1, *Tg*CerS2 and *Tg*UPRT ORFs.

Primer name	Forward sequence	Reverse sequence	Method
*Tg*CerS1 sgRNA1	ACACGTTCCACGGGTTTTAGAGCTAG AAATAG	AACTTGACATCCCCATTTAC	Q5
*Tg*CerS1 sgRNA2	CGCTCACCAACGCGTTTTAGAGCTAG AAATAG	AACTTGACATCCCCATTTAC	Q5
*Tg*CerS2 sgRNA1	GACGTCATAGGTTCTCGTTTTAGAGC TAGAAATAG	AACTTGACATCCCCATTTAC	Q5
*Tg*CerS2 sgRNA2	GAGACGACGTCATAGGTTTTAGAGCT AGAAATAG	AACTTGACATCCCCATTTAC	Q5

Table S5. List of primers used to create GFP donor cassette. Each pair of the donor DNA primers represents 50bp flanking omology (plus overlapping region to GFP) with which the GFP cassette was amplified to undergo homologous recombination after genomic DNA was cut by CRISPR.

Primer name	Forward sequence	Reverse sequence
TgCerS 1.GFP	TCAGCGCCTTCAAATACTTCGGCCGGA AATTTGACATTGTGAAGCCGGGGGGT ACCGGGCCCCCCCTC	CGAAAACTGGGGGTCCCTTACCACAGA TTCTCTGCGAATTTGCAAAGCTTTTAAT TAACTGCAGCTTTG
TgCerS 1.GFP	ATTCACCAAGGCGAAAGTGGAGTCGTT GATCTCTACCTCCTCAATCCGACGGGT ACCGGGCCCCCCCTC	TGAGGAAGGCGCTTACCAAGACGACG AAGTGGACATCTGCCCAACCGAGATTA ATTAACTGCAGCTTTG
TgCers 2.GFP	CTACATCAAGGACCGCGTTGCAGACCG ACAGAAAACTTTCGCCGCCTATCGGGT ACCGGGCCCCCCCTC	ACAGGCTCCTGCGATGAGGGCCGAGG CCGAGACGACGTCATAGGTTCTCGTTA ATTAACTGCAGCTTTG
TgCers 2.GFP	TCAAGGACCGCGTTGCAGACCGACAGA AAACTTTCGCCGCCTATCCGAGAGGGT ACCGGGCCCCCCCTC	ACGAGACAGGCTCCTGCGATGAGGGC CGAGGCCGAGACGACGTCATAGGTTTA ATTAACTGCAGCTTTG

Table S6. Cloning primers for diCre:*Tg*CerS1 plasmid construct.

Primer name	Forward sequence	Reverse sequence	Method
F01 (5' UTR – loxP1) CerS1	TGGCCGATTCATTAATGCAGGGTGT GCCAGGGTTTAGAGG	CAGGCGTCATTTTGTCGGAAT TCTATAACTTCGTATAATGTAT GCTATACGAAGTTATCGTCCC CACTTCTAAATGGAGC	In-Fusion cloning
F02 CerS1 ORF – 2A	CGACAAAATGACGCCTGTTCCCTAC CAG	TATGGACCTCAGGTCCAGGGT TCTCTTCGACGTCTCCGCAAG TGAGGAGGCTGCCTCGTCCTT CTCCCCGGGTTTTGCACTTTG	In-Fusion cloning
F03 Ty.KillerRED.lox P.YFP-5'UTR.HXGPRT	CCCTGGACCTGAGGTCCATACTAAC CAAGATCCAC	AAGGAAAAAATTACTTCTCGA ACTTTTTGCGA	In-Fusion cloning
F04 (3' UTR) CerS1	CGAGAAGTAATTTTTTCCTTCTCCTT GCACTTTGG	CCTCTTCGCTATTACGCCAGG TCTTTCGGATTGGAAGCGTTT GG	In-Fusion cloning

Table S7. Cloning primers for diCre:*Tg*CerS2 plasmid construct.

Primer name	Forward sequence	Reverse sequence	Method
F01 (5' UTR – loxP1) CerS2	TGGCCGATTCATTAATGCAGAAGCT TGTCTCTGTCAAGTG	CCAACTTCATCATTTTGTCGGAAT TCTATAACTTCGTATAATGTATGCT ATACGAAGTTATTTTGACGGAAGT CGGGAGAAG	In-Fusion cloning
F02 CerS2 ORF – 2A	CGACAAAATGATGAAGTTGGATCCG TTTTTCACC	TATGGACCTCAGGTCCAGGGTTC TCTTCGACGTCTCCGCAAGTGAG GAGGCTGCCTCGTCCTTCTCCGT GGCGCTTCTTGCTATCG	In-Fusion cloning
F03 Ty.KillerRED.loxP.YFP-5'UTR.HXGPRT	CCCTGGACCTGAGGTCCATACTAAC CAAGATCCAC	CTCTTTCCTTTTACTTCTCGAACTT TTTGCGA	In-Fusion cloning
F04 (3' UTR) CerS2	CGAGAAGTAAAAGGAAAGAGTGTGT AGGCG	CCTCTTCGCTATTACGCCAGTTGC GGTCTCTTTCTCTCGC	In-Fusion cloning

Table S8. List of primers used to intentify and confirm contitional mutant strains of RH.diCre:*Tg*CerS1.

Primer Name	Genomic locus target	Forward sequence	Reverse sequence
dCS10-11	Ki.Red	ATGGGTTCAGAGGGCGGC	ATGCATATCCTCGTCGCTAC
dCS12-13	TGGT1_316450	ATGCCGGAGGAGTTGCGGAGATT CGAGG	CCCGGGTTTTGCACTTTGAATTCCCC CC
dCS14-15	EYFP	ATGGTGAGCAAGGGCGAGGAG	TTACTTGTACAGCTCGTCCATGC
dCS16-17	HXGPRT	ATGGCGTCCAAACCCATTGA	TTACTTCTCGAACTTTTTGCGAG
dCS18-19	5'UTR-loxP-*Tg*Cers1-2A-KillerRed-loxP-EYFP-3'UTR-5'UTR-HXGPRT-3'UTR	CTCTCACGTTTTGCGCCTCAAGA GGAAC	CGATCAAGCGTGCGTTCTTTTCTGTT TT
dCS20-21	5'UTR-3'UTR	ATTCTTCTGGTTCCTCGTAGATTA TAGTTCCC	CGATGTATCTGATAACAAAGTGACCC TACA

Table S9. List of primers used to intentify and confirm contitional mutant strains of RH.diCre:*Tg*CerS2.

Primer Name	Locus target	Forward sequences	Reverse sequence
dCS24-25	5'UTR-TGG1_283710	CCTCATACTTCTGCACATCCGC	GCAGCATCTTCTCTGCCAGCG
dCS26-27	HXGPRT-3'UTR	ATGTTCCGCGACTTCGACCACG	CGCTCCATTGCCTCGAGTTTGC
dCS28-29	5'UTR-loxP-TgCers2-2A-KillerRed-loxP-EYFP-3'UTR-5'UTR-HXGPRT-3'UTR	GCCTACACACTCTTTCCTTTTAAG TGG	TGTCTTCTTTCTCCTCGTTCGTCCC
dCS30-31	5'UTR-3'UTR	CCGTTCGAGTGTCGCCTGAACTG TTGTC	GTTTTGCGTCGGGTCTCTTCTCTGTT TTC

Table S10. Species name, accesion numbers and online databases sources for sequences used in analyses.

No	Label	Species name	Accession Number	Database Source
1	*AtCerS1*	*Arabidopsis thaliana*	sp|Q6NQI8	https://www.uniprot.org
2	*AtCerS2*	*Arabidopsis thaliana*	sp|Q9LDF2	https://www.uniprot.org
3	*AtCerS3*	*Arabidopsis thaliana*	sp|Q9LJK3	https://www.uniprot.org
4	*CycCerS2*	*Cyclospora cayetanensis*	cyc_00898	https://toxodb.org/toxo/app/
5	*EthCerS2*	*Eimeria tenella*	ETH_00025685	https://toxodb.org/toxo/app/
6	*HhaCerS2*	*Hammondia hammondi*	HHA_283710	https://toxodb.org/toxo/app/
7	*TgCerS2*	*Toxoplasma gondii*	TGGT1_283710	https://toxodb.org/toxo/app/
8	*Csui*CerS2	*Cystoisospora suis*	CSUI_005129	https://toxodb.org/toxo/app/
9	Sn3Cer2	*Sarcocystis neurona*	SN3_01001010	https://toxodb.org/toxo/app/
10	*Pf*CerS2	*Plasmodium falciparum*	PF3D7_0508200	https://plasmodb.org/plasmo/app/
11	*Pf*CerS1	*Plasmodium falciparum*	PF3D7_1403700	https://plasmodb.org/plasmo/app/
12	*Cmu*CerS2	*Cryptosporidium muris*	CMU_042100	https://cryptodb.org/cryptodb/app/
13	*Hs*CERS1	*Homo sapiens*	HsCERS1 - sp|P27544	https://www.uniprot.org
14	*Hs*CERS2	*Homo sapiens*	HsCERS2 - sp|Q96G23	https://www.uniprot.org
15	*Hs*CERS3	*Homo sapiens*	HsCERS3 - sp|Q8IU89	https://www.uniprot.org
16	*Hs*CERS4	*Homo sapiens*	HsCERS4 - sp|Q9HA82	https://www.uniprot.org
17	*Hs*CERS5	*Homo sapiens*	HsCERS5 - sp|Q8N5B7	https://www.uniprot.org
18	*Hs*CERS6	*Homo sapiens*	HsCERS6 - sp|Q6ZMG9	https://www.uniprot.org
19	*Cf*CerS1	*Cytauxzoon felis*	CF001484	https://piroplasmadb.org/piro/app/
20	*Ta*CerS1	*Theileria annulata*	TA12945	https://piroplasmadb.org/piro/app/
21	*Cyc*CerS1	*Cyclospora cayetanensis*	cyc_05293	https://toxodb.org/toxo/app/
22	*Sn3*CerS1	*Sarcocystis neurona*	SN3_01900490	https://toxodb.org/toxo/app/
23	*Hha*CerS1	*Hammondia hammondi*	HHA_316450	https://toxodb.org/toxo/app/
24	*Tg*CerS1	*Toxoplasma gondii*	TGGT1_316450	https://toxodb.org/toxo/app/
25	NclivCerS1	*Neospora canium*	NCLIV_058920	https://toxodb.org/toxo/app/
26	*Csui*CerS1	*Cystoisospora suis*	CSUI_001693	https://toxodb.org/toxo/app/
27	*Cmu*CerS1	*Cryptosporidium muris*	CMU_034340	https://cryptodb.org/cryptodb/app/

REFERENCES

1. Jumper, J., Evans, R., Pritzel, A., Green, T., Figurnov, M., Ronneberger, O., Tunyasuvunakool, K., Bates, R., Zidek, A., Potapenko, A., Bridgland, A., Meyer, C., Kohl, S. A. A., Ballard, A. J., Cowie, A., Romera-Paredes, B., Nikolov, S., Jain, R., Adler, J., Back, T., Petersen, S., Reiman, D., Clancy, E., Zielinski, M., Steinegger, M., Pacholska, M., Berghammer, T., Bodenstein, S., Silver, D., Vinyals, O., Senior, A. W., Kavukcuoglu, K., Kohli, P., and Hassabis, D. (2021) Highly accurate protein structure prediction with AlphaFold. *Nature* **596**, 583-589
2. Yang, J., Anishchenko, I., Park, H., Peng, Z., Ovchinnikov, S., and Baker, D. (2020) Improved protein structure prediction using predicted interresidue orientations. *Proc Natl Acad Sci U S A* **117**, 1496-1503
